# The Interplay between Alpha-Synuclein Clearance and Spreading

**DOI:** 10.3390/biom5020435

**Published:** 2015-04-14

**Authors:** Tomás Lopes da Fonseca, Anna Villar-Piqué, Tiago Fleming Outeiro

**Affiliations:** 1 Department of Neurodegeneration and Restorative Research, Center for Nanoscale Microscopy and Molecular Physiology of the Brain, University Medical Center Göttingen, Göttingen 37073, Germany; E-Mails: tlopesdafonseca@gmail.com (T.L.F.); avillar@gwdg.de (A.V.-P.); 2 Instituto de Fisiologia, Faculty of Medicine, University of Lisbon, Lisboa 1649-028, Portugal; 3 CEDOC, Faculdade de Ciências Médicas, Universidade Nova de Lisboa, Lisboa 1150, Portugal

**Keywords:** alpha-synuclein, Parkinson’s Disease, autophagy, spreading, prion

## Abstract

Parkinson’s Disease (PD) is a complex neurodegenerative disorder classically characterized by movement impairment. Pathologically, the most striking features of PD are the loss of dopaminergic neurons and the presence of intraneuronal protein inclusions primarily composed of alpha-synuclein (α-syn) that are known as Lewy bodies and Lewy neurites in surviving neurons. Though the mechanisms underlying the progression of PD pathology are unclear, accumulating evidence suggests a prion-like spreading of α-syn pathology. The intracellular homeostasis of α-syn requires the proper degradation of the protein by three mechanisms: chaperone-mediated autophagy, macroautophagy and ubiquitin-proteasome. Impairment of these pathways might drive the system towards an alternative clearance mechanism that could involve its release from the cell. This increased release to the extracellular space could be the basis for α-syn propagation to different brain areas and, ultimately, for the spreading of pathology and disease progression. Here, we review the interplay between α-syn degradation pathways and its intercellular spreading. The understanding of this interplay is indispensable for obtaining a better knowledge of the molecular basis of PD and, consequently, for the design of novel avenues for therapeutic intervention.

## 1. Introduction

### 1.1. Parkinson’s Disease

Parkinson’s disease (PD) is the most common neurodegenerative disorder with movement impairment. At the clinical level, the disease is classically characterized by resting tremor, bradykinesia, postural instability, and muscular rigidity [1]. Although PD was initially classified as a movement disorder, it is now well accepted that non-motor symptoms precede and follow motor disabilities. Thus, PD is currently regarded as a disorder affecting the whole-brain [2]. In fact, hyposmia is one of the prevalent symptoms in early stages of the disease, with several olfactory-related brain areas found severely affected in PD patients [3,4,5,6,7,8].

Pathologically, PD is characterized by the degeneration of dopaminergic neurons in the *substantia nigra pars compacta*, and the accumulation of intracellular, proteinaceous inclusions in the surviving neurons, known as Lewy bodies (LBs) and Lewy neurites (LNs) [1].

According to Braak’s staging theory, the evolution of LB pathology is thought to initiate in either the lower brainstem or in the olfactory bulb. Then, as the disease progresses, LB pathology appears in other brain areas, including the cerebral cortex, impacting different neural networks and leading to non-motor symptoms such as depression, cognitive decline and hallucination episodes [4,9,10,11]. Defining the responsible mechanism for PD pathology progression has challenged the scientific community for almost 200 years. During the last decade, the “prion-like” hypothesis has gained particular emphasis based on findings from independent experiments in PD patients. In patients that received grafts of normal fetal mesencephalic brain tissue, a time-dependent accumulation of LBs and reduced immunostaining for dopamine transporter was observed [12,13,14,15,16].

Only a minority of PD cases have been linked to genetic factors (less than 10%) with the majority being considered sporadic, of unknown origin. Until now, more than 20 genes have been associated with PD [17], but this number is expected to increase as new studies including larger number of patients are conducted. The first gene linked to familial forms of PD was *SNCA*, encoding for the protein alpha-synuclein (α-syn). Thus far, mutations, as well as duplication or triplications of the *SNCA* gene, are associated with familial forms of PD [18,19,20,21,22,23,24,25]. Additionally, recent studies also revealed that polymorphisms in the *SNCA* gene lead to increased risk for developing PD [26,27,28,29]. Thus, for all of the above, α-syn is regarded as one of the major culprits in both genetic and idiopathic forms of PD.

### 1.2. Alpha-Synuclein

α-syn is a small 14.5 kDa protein, initially found to be located to pre-synaptic terminals and in the cell nucleus [30]. α-syn consists of 140 amino acids that can be divided into three domains that confer unique properties to the protein. The N-terminal region (residues 1–60) contains several imperfect repeats of a consensus motif (KTKEGV). Due to this repeated motif, also found in Apolipoprotein A–I, that can form amphipathic alpha-helices upon lipid-binding [31], this region confers membrane binding ability to α-syn [32,33]. Interestingly, all six PD-related mutations described thus far are located in this domain [18,19,20,21,22,23]. The central region, consisting of residues 61 to 95, is commonly known as the non-Aβ component of plaque (NAC) domain. This highly hydrophobic region contains 12 amino acids (71–82) essential for α-syn filament formation [34]. The last 44 amino acids of α-syn confer negative charge to the protein due to abundance of glutamates and aspartates. These negative residues appear to have an important effect in modulating α-syn aggregation [35] and, in fact, this domain is essential for the formation of calcium-mediated annular oligomers via direct binding [36,37]. In addition, the C-terminus of α-syn has been suggested to play a role on the protein’s chaperone-like activity [38].

For years, α-syn was regarded as a natively unfolded, monomeric protein. Recently, some studies proposed it may occur as a natively folded helical tetramer [39,40,41], but this subject remains highly controversial [42]. Nevertheless, the possibility that α-syn occurs naturally in a folded state brought new perspectives into the process of its oligomerization and aggregation, one of the major mysteries in the field [17]. Major efforts have been placed in understanding which form of α-syn acts as the toxic species but consensus has not been reached. Currently, it is generally accepted that smaller oligomeric species are more toxic than larger aggregated forms [43,44,45]. However, some authors still claim that the final mature aggregates are the most dangerous for cell homeostasis [46,47].

α-syn is prone to several types of posttranslational modifications (PTMs). Ubiquitination [48,49,50,51], sumoylation [52,53,54] and N–terminal acetylation [55,56,57] have been described. In addition, α-syn can be phosphorylated [58] in two serines (S129 and S87) and three tyrosines (Y125, Y133 and Y135). It is estimated that approximately 90% of the α-syn present in LBs is phosphorylated in S129 [59]. Several kinases, including CKs, PLKs and GRKs, can phosphorylate α-syn on S129 [58,60,61,62,63,64,65]. Interestingly, some of those enzymes were found up-regulated in PD brains [63] or present in LBs [61,66]. S129 phosphorylation can inhibit α-syn fibrillization [67,68], and a similar effect was observed for S87 [69]. Unfortunately, the full functional relevance of phosphorylation in both physiological and pathological contexts is elusive [70,71,72].

In contrast, nitration of tyrosine residues in α-syn (Y39, Y125, Y133, Y136) is known to produce toxic effects. In particular, nitrated α-syn is present in LBs [73] and nitrated α-syn oligomers promote mitochondrial impairment and cell death in mammalian cell culture [74]. Furthermore, administration of nitrated α-syn in the *substantia nigra* of rats induces severe dopaminergic neuronal cell death, and the down-regulation of striatal dopamine and dopamine receptor D2 [75]. Nitration on Y39 blocks α-syn fibril formation and reduces monomer degradation via the ubiquitin proteasome system [76].

Finally, truncated α-syn is found in LBs and in animal models and it is thought that some familial α-syn mutations might promote its truncation [48,77,78]. *In vitro*, C-terminally truncated α-syn enhances fibril assembly and induces fibril formation of full-length α-syn [79,80]. Nevertheless, the opposite effect was reported upon calpain1 or neurosin mediated truncation [81,82,83,84]. Interestingly, these two proteases cleave α-syn near the NAC domain highlighting the importance of this region on α-syn aggregation.

## 2. Protein Degradation Systems

Proper protein degradation is a crucial process in intracellular homeostasis and it is ensured by two independent, but complementary, systems that work in symbioses. These two pathways, the Autopaghy-Lysosomal Pathway (ALP) and the Ubiquitin Proteasome System (UPS), are named upon their final destinations, the lysosome and the proteasome, respectively. Monomeric α-syn can be actively degraded by both organelles [85,86] that compensate each other upon one’s failure [87]. When it comes to eliminating higher molecular species, including oligomers and aggregates, the burden shifts to the lysosome [88]. Therefore, and considering the purpose of this review, we will focus specifically on the pathways culminating in the lysosomal compartment.

Autophagy (meaning “self-eating”, in Greek) consists in the process of decomposition and degradation of cellular components and organelles via the lysosomal compartment. Autophagy itself serves two main purposes: the clearance of deleterious intracellular components, and the recycling of macromolecules from functional pre-existing organelles and proteins to guarantee proteome renewal [89].

Depending on the cargo delivery method, autophagy can be divided in three main types: chaperone-mediated autophagy (CMA), macroautophagy and microautophagy. To our knowledge, there is presently no evidence linking α-syn to microautophagy. Thus, here we focus only on CMA and macroautophagy.

### 2.1. α-Syn and CMA—A Symbiotic Relation with a “Do not Disturb” Sign

**Figure 1:**
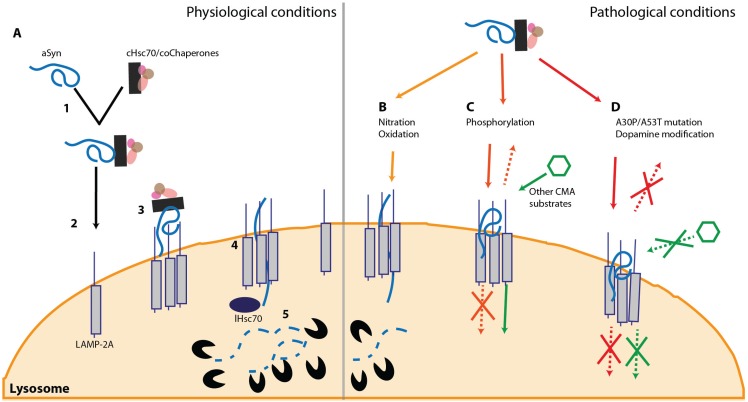
α-syn and the CMA. (**A**) Under physiological conditions, Hsc70 recognizes the KFERQ domain of α-syn (1) and targets the protein towards the lysosome (2). At the lysosomal membrane, α-syn interacts with LAMP-2A and promotes its oligomerization (3) leading to the entrance of the protein into the lysosome (4). Once inside the lysosome, α-syn is degraded by proteases (5); (**B**) PTMs such as oxidation and nitration slightly inhibit this pathway and reduce α-syn degradation; (**C**) Phosphorylation of α-syn on S129 impairs its degradation via CMA. However, while the phosphorylated form of protein does not block this pathway (**D**), dopamine-modified α-syn and some familial mutations (A30P and A53T) that are also not degraded via CMA, can block this pathway and prevent the degradation of other CMA substrates.

CMA is a particular cellular mechanism for protein degradation linked to the lysosome. Unlike other degradation systems, CMA is based on the recognition of a specific amino acid sequence (KFERQ) [90]. This motif is found in nearly 30% of cytoplasmic proteins, including α-syn, and in some compartment-associated proteins [90]. Protein misfolding, partial folding or PTMs, such as phosphorylation and acetylation, can promote or inhibit the CMA pathway [91]. Mechanistically, CMA relies on the proper identification and binding of cytosolic Hsc70 (cHsc-70) to the target substrate [92]. This complex is later directed to the lysosomal membrane where it interacts with the lysosome-associated membrane protein type 2a (LAMP2A) [93,94]. The final step of this translocation process requires the lysosome-associated Hsc70 (lHsc70) that targets the substrate to degradation (Figure 1A) [95].

The reciprocal interaction between α-syn and CMA protein degradation has been of high interest in the last decade. Using *in vitro* purified lysosomes from liver, it was initially described that α-syn can be actively degraded via CMA and, more interestingly, that two α-syn familial mutations, A30P and A53T, impair CMA degradation (Figure 1D). Both α-syn mutants exhibited higher affinity to the LAMP2A receptor, blocking CMA at the LAMP2A level which, ultimately, leads to a full impairment of the pathway [85]. Later on, it was shown that α-syn can be degraded through this pathway, in several cell culture models [96]. In mice, increase of both LAMP2A and Hsc70 levels was observed upon overexpression of wild-type (wt) α-syn [97]. In a comprehensive study on α-syn PTMs, a slight inhibition of CMA was observed upon nitration and oxidation (Figure 1B). Furthermore this pathway is stalled when α-syn is phosphorylated (Figure 1C) or exposed to dopamine. Interestingly, just the latter can also completely block the CMA degradation machinery (Figure 1D) [98]. The previous report also proved that CMA is only capable of degrading monomers and dimers of α-syn, but not oligomers [98].

In addition to the direct effect of α-syn on CMA, this interplay may also impact other degradation pathways. In particular, the A53T α-syn mutation can block CMA leading to an activation of macroautophagy and an increase of toxicity cultured cells [99].

The protective effect of functional CMA on decreasing α-syn toxicity and aggregation was confirmed *in vivo* by overexpression of LAMP2A [100]. Furthermore, LAMP2A down-regulation is the main cause of decreased CMA activity observed in ageing [101] and, together with Hsc70, it is down-regulated in the *substantia nigra* and amygdala from PD patients [102]. Nevertheless, some studies describe the existence of an alternative LAMP2A-independented CMA pathway in fibroblasts [103].

Recently, microRNAs (miRNAs) gained attention also in the context of α-syn degradation via CMA. Interestingly, miRNAs have been linked to PD [104], and several miRNAs targeting the CMA pathway (LAMP2A or Hsc70) have been described. The majority was found to impair α-syn degradation and potentially alter the aggregation state of the protein [105,106]. In an initial report, four miRNAs were found to reduce LAMP2A levels while three others directly down-regulated Hsc70. In all cases an increase in α-syn was observed. Moreover, these 7 miRNAs were found to be up-regulated in the *substantia nigra* from PD patients [106], correlating with a decrease in both CMA related protein levels. A more recent study identified another miRNA, miRNA320a, although it had been previously discarded [105,106]. This particular miRNA320a has been linked to a panoply of diseases ranging from fibromyalgia [107] to Waldenström macroglobulinemia [108] and more recently to the Ebola virus [109].

### 2.2. Macroautophagy of α-Syn—The Last Resource to Avoid Protein Aggregation

**Figure 2:**
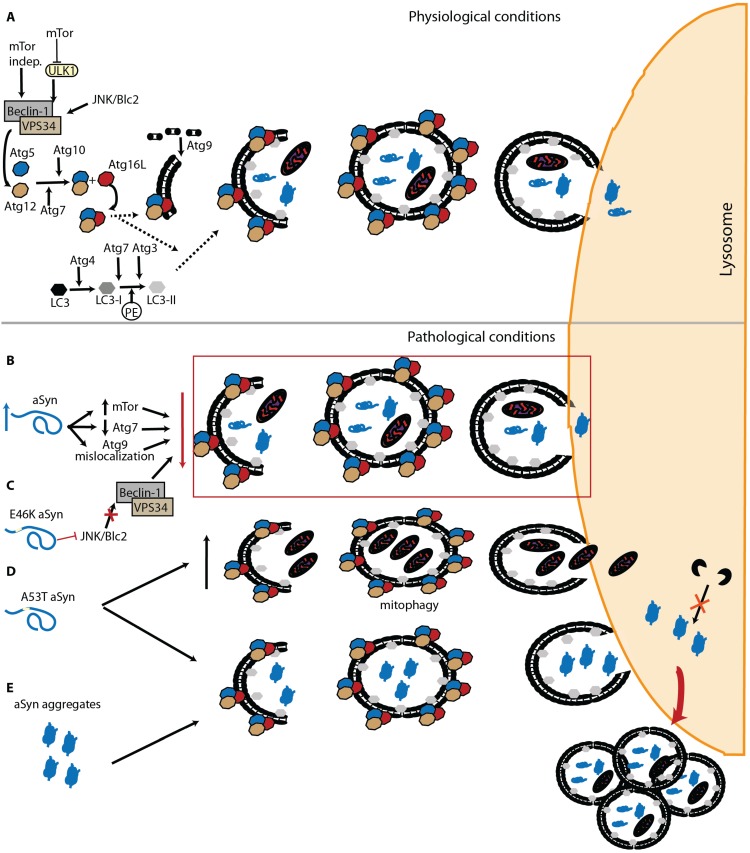
α-syn and Macroautophagy. (**A**) Macroautophagy is composed of fine-tuned machinery that ensures specific target recognition and cargo delivery to the lysosome; (**B**) Accumulated α-syn increases mTor and decreases Atg7 levels, promoting mislocalization of Atg9 and leading to impairment of macroautophagy; (**C**) The α-syn familial mutation E46K can inhibit macroautophagy via JNK/Blc2, an mTor independent pathway; (**D**) On the other hand, two different effects are associated with the A53T α-syn mutation: an increase in mitophagy, and accumulation of autophagosomes due to impaired degradation; (**E**) α-syn aggregates cannot be degraded by macroautophagy, leading to the impairment of the pathway.

Macroautophagy, commonly referred to as “autophagy”, is the most scrutinized and well known of the three autophagic mechanisms. This “content-blind” pathway relies on the formation of *de novo* double membrane-bound vesicles to sequester intracellular components, including whole organelles, towards the lysosome [110,111]. This membrane formation mainly relies on the autophagy related protein (Atg) 9, both in yeast and humans [112,113,114,115]. Macroautophagy is found constitutively active but further activation via the mTOR pathway, the mammalian target of rapamycin [116], or the PI3kinase/beclin/vsp34 pathway, also known as mTOR-independent pathway, is possible [117]. Autophagosome formation requires two ubiquitination steps highly regulated by Atg proteins [118,119,120]. Initially Atg12 is conjugated with Atg5, a process involving Atg7 and Atg10 [121,122]. The Atg12-Atg5 complex is later targeted to the autophagosome with Atg16 [123,124]. The second ubiquitination step requires Atg8 (also known by LC3). LC3 is C-terminally cleaved by Atg4 to form LC3-I [125,126], which is then conjugated to the lipid phosphatidylethanolamine (PE) by Atg7 and Atg3 to generate LC3-II [122,127]. Interestingly, the Atg12-Atg5 complex originated from first ubiquitination seems to be necessary for the LC3 processing and localization at the autophagosome membrane (Figure 2A) [128,129].

Depending on the cargo being degraded, alternative players can be involved in the task. In the case of protein aggregates, an alternative has been reported and is usually referred to as aggrephagy. Mechanistically, aggrephagy can be divided in two pathways: HDAC6, or BAG-3 mediated. The first requires the addition of lysine (K) 63-poliubiquitination chains to the substrate. This can be achieved with the involvement of the PD-related protein Parkin [130]. HDAC6 can then recognize the substrate and translocate it, in a microtubule-dependent manner, towards the aggregosome [130,131]. Once in the aggregosome, the aggregate degradation requires the intervention of p62 and NBR1 that can work separately or possibly in a duet, since they can interact [132]. Importantly, both proteins interact with PE-LC3, an essential component of the autophagosome membrane [133,134,135]. Both p62 and NBR1 also interact with K63-poliubiquitinated chains suggesting that this PTM could be essential for HDAC6-mediated aggrephagy [135,136,137].

The second mechanism, mediated by BAG-3, also involves p62 and NBR1, but differs in the initial steps of the cascade. The process starts with the “labeling” of the cargo with Hsc70, a reaction that requires both BAG-3 and CHIP [138]. Interestingly CHIP has been repetitively associated with α-syn homeostasis [51,139,140]. This pathway appears to be ubiquitin-independent [138] but, considering that the end targets are also p62 and NBR1, it is possible that the substrates are also ubiquitinated along the way.

Several proteins involved in aggrephagy are common to other cellular mechanisms. In particular, HDAC6 has been extensively associated with multivesicular bodies (MVB) and with the delivery of their cargo to lysosomes or to the extracellular environment, via exosomes [141]. In any case, we still have to understand what directs the proteins towards one of the pathways and, in the future, how this can be modulated to enhance the cellular response under stress conditions, such as in the presence of protein aggregation.

α-syn degradation via macroautophagy has been mostly studied using specific inhibitors and enhancers of this pathway. Both in cell culture or *in vivo*, wt and mutated α-syn can be degraded through macroautophagy, a process that may be at least partly modulated by Beclin-1 [142,143,144]. Furthermore, blockade of this pathway generally leads to the accumulation of high molecular weight species of α-syn, although different macroautophagy inhibitors reveal distinct outcomes [88,96]. Another line of evidence indicates that macroautophagy inhibitors affect exclusively mutant A53T α-syn, while other studies describe different behaviors that vary according to the selected inhibitor [102,145]. In yeast, an organism lacking CMA, our group recently demonstrated that α-syn phosphorylation on S129 can modulate the protein degradation via macroautophagy and, ultimately, inclusion formation [68]. In yeast, it was also described that the interplay between phosphorylation and sumoylation has a direct impact on the autophagic degradation of α-syn [54]. In general, it is believed that macroautophagy is crucial for the lysosomal degradation of oligomeric and aggregated α-syn, since CMA is unable to handle large protein species [98,146]. Once inside the lysosome, α-syn is mainly degraded by Cathepsin D (CTSD) [147]. In cells, overexpression of an inactive mutant of this aspartyl protease leads to increased α-syn levels [148] while CTSD knockout mice exhibited accumulation of higher molecular weight α-syn species [149].

Interestingly, α-syn can also have a direct impact on this lysosomal degradation pathway. α-syn overexpression can inhibit macroautophagy via an interaction with Rab1a that culminates in a mislocalization of Atg9 [150]. Furthermore, an increase in mTor and decrease in Atg7 levels has been observed in both Dementia with LB (DLB) patients and α-syn transgenic mice (Figure 2B) [151]. The same study also reported the presence of enlarged autophagosomes and lysosomes, as observed in cells overexpressing α-syn [142]. In a recent work, α-syn aggregates were able to resist macroautophagy leading to a failure of the pathway and accumulation of autophagosomes (Figure 2E) [47].

In addition to the effects of wt α-syn on autophagy, mutations associated with familial forms of PD also seem to differentially affect this protein degradation pathway. In the E46K α-syn mutant, an impairment of autophagy is observed, via inactivation of the JNK1/Blc2 pathway (Figure 2C) [152]. While the A53T mutation seems to promote increased mitophagy [153] and the accumulation of autophagosomes due to impaired degradation (Figure 2D) [154].

In summary, and despite conflicting evidence, it seems unquestionable that there is a strong interplay between α-syn and autophagy pathways, and that this may enable therapeutic interventions.

## 3. Prion-Like Spreading of α-Syn Pathology

For the last decade, it has been postulated that neurodegeneration-related proteins, which are usually present in an abnormal aggregated state, can intercellularly transmit their abnormal conformation to homotypic native proteins. Here, we focus on the concept of spreading in the context of the overall cellular protein homeostasis. In this regard, protein degradation is a key mechanism that undoubtedly influences α-syn propagation, although its precise modulatory effect is still unclear.

### 3.1. Insight from Pathological Studies

The systematic study of post-mortem human brain tissue from PD patients suggested that α-syn pathology progresses throughout the brain in a pattern that follows the progression of clinical symptoms. This is known as Braak’s staging for PD [4] and is now considered as the first evidence of the hypothesis of the prion-like spreading of α-syn pathology. Although Braak’s PD staging could be merely due to the differential disease susceptibility among distinct brain regions, the stereotypic pattern and location of the zones affected in each stage suggests a spatial-temporal dissemination of the α-syn pathology, which indeed correlates with cognitive decline [155]. From the first regions affected, the olfactory bulb and brainstem, α-syn pathology spreads caudo-rostrally to susceptible midbrain regions and to subcortical and cortical areas as PD progresses [4]. The initial sites where α-syn lesions appear led Braak and co-workers to postulate the theory that indeed sporadic α-syn pathology is initiated by an exogenous pathogenic agent [156,157], although experimental evidence for such agent is still lacking.

The discovery of LBs in young neurons that were grafted into the brains of PD patients boosted the idea of the prion-like spreading of pathology in the brain [15,16]. The grafted cells were obtained from human embryos and the transplantation was performed only 11–16 years before the pathological examination of the brains, upon death of the patients. Since this period of time is extremely short for α-syn pathology to spontaneously develop under normal conditions [15,16,158], these findings suggested that α-syn pathology could be transmitted from diseased to healthy cells. Among the distinct agents and factors that could be responsible for transmitting the pathology, the misfolded/aggregated state of α-syn was a strong contender [159], establishing the basis for the current hypothesis of the prion-like spreading of α-syn pathology. The α-syn that accumulated in the grafted neurons displayed the same characteristics of LBs and LNs in the host tissue [160], suggesting a common gradual formation mechanism. In addition, those cells were also described to suffer a degenerative process and loss of dopaminergic phenotype [161,162]. The presence of LBs in young grafted neurons has been repeatedly confirmed in experiments in rodents overexpressing human α-syn. In both mice [163] and rats [164,165], the time-dependent transfer of α-syn from the host to the graft was demonstrated by the presence of human α-syn puncta in the transplanted cells. In rats, the inclusions formed were described to consist of a core of human α-syn surrounded by endogenous rat α-syn, clearly suggesting a seeding mechanism [164].

Another relevant finding was the discovery of α-syn in human body fluids, such as cerebrospinal fluid (CSF) and blood plasma [166,167]. Taking into account that α-syn is traditionally considered a cytoplasmic protein, its extracellular presence could be associated to passive release caused by cell death and neurodegeneration. However, the similar levels of monomeric α-syn found in the CSF and plasma of PD and control individuals hinted at a physiological secretion process [166,167]. Unfortunately, there is still no consensus on the role of extracellular α-syn and whether this is related to pathological conditions. In an attempt to clarify this issue, some studies suggest the occurrence of increased levels of the α-syn oligomeric fraction in the CSF of patients with synucleinopathies [168,169].

Overall, these studies indicate that, on one side, there is a fraction of extracellular α-syn present in physiological conditions and, on the other, that there might be an increase in extracellular α-syn oligomeric species in diseased individuals. This needs to be further confirmed but requires the development of novel and better tools, such as antibodies that recognize specific types of α-syn species and/or conformers.

### 3.2. The Proof of Concept: in Vivo α-Syn Propagation Experiments

The first *in vivo* α-syn propagation studies were performed in host animals already susceptible to an age-dependent synucleinopathy. Brain homogenates extracted from symptomatic transgenic mice expressing human A53T α-syn triggered an early onset of the pathology with expected clinical and biochemical hallmarks, when injected into the brains of young asymptomatic mice [170,171]. The same result was obtained when using recombinant human α-syn fibrils assembled *in vitro* [170]. This finding was crucial to demonstrate that α-syn pathology can be accelerated and spread through the brain after inoculation with pathological material. The acceleration of the pathology was interpreted as a seeding effect of the exogenous material over the otherwise slow and age-dependent aggregation of the endogenously expressed α-syn.

Subsequent experiments provided additional insight into the process. Injecting recombinant mouse α-syn fibrils in the dorsal striatum of young wt mice induced pathological α-syn transformation leading to motor deficits [172]. Similar induction of α-syn pathology was described in another study comparing the effect of recombinant human α-syn and of insoluble brain fractions extracted from DLB patients [173]. α-Syn accumulation was described in several brain regions distant from the injection site, demonstrating the propagation capacity of the injected material [172,173]. In addition to the biochemical hallmarks, the exogenous induction of α-syn pathology also includes a progressive nigrostriatal neurodegeneration in the recipient animals as demonstrated by the injection of PD brain extracts into the brains of wt mice [174]. Data derived from experiments performed with recombinant protein or with brain-derived pathological samples should be interpreted in a complementary way. On one hand, using brain homogenates demonstrates that biological protein material is able to induce homotypic pathology in another organism, a primary characteristic of prion diseases. On the other hand, experiments using recombinant α-syn fibrils enable the confirmation that α-syn is the major component for pathology induction. Interestingly, it was demonstrated that mouse α-syn fibrils displayed a higher efficiency in inducing a synucleinopathy in wt mice than human α-syn fibrils, suggesting the existence of a species barrier effect [173,175]. Finally, similar experiments were performed in monkeys injected with LB-fractions derived from sporadic PD brains, demonstrating that the induction of a synucleinopathy also occurs in non-human primates and that, in the context of propagation of α-syn pathology, the monkey-human species barrier is, at best, rather soft [174].

Despite existing discrepancies regarding the methodologies used in different works [176], and the number of open questions that need to be answered [177], the recent studies demonstrate that synucleinopathies can be induced by external agents, which raises the issue of whether these neurological disorders can also have an infectious origin, even if they do not appear to be contagious. 

### 3.3. Mechanisms of α-Syn Cell-to-Cell Propagation

Although the prion-like spreading of α-syn pathology hypothesis is becoming widely accepted, little is still known about the molecular mechanisms underlying this process. It is possible that the mechanisms of α-syn propagation are probably dependent on the cell type, their physiological state, and the type of α-syn species transmitted. The various possible mechanisms have been extensively reviewed by many in the field [17,178].

The spreading of α-syn pathology is due to the cell-to-cell transmission of pathological forms of α-syn. This process requires at least: (i) α-syn release from a cell; (ii) α-syn uptake by a neighboring cell; and (iii) α-syn pathology induction in the recipient cell probably caused by seeding of the endogenous α-syn [178]. The passive release of α-syn from dying cells could be a logical possibility, due to the ongoing neurodegenerative process. However, the lack of a clear increase in α-syn CSF levels between diseased and control individuals [179] limits the likelihood of this process as the main mechanism of release.

Given the membrane-binding capacity of α-syn, another possibility is that secretion might take place through an unconventional ER/Golgi-independent secretion pathway [180], or exosome-mediated pathway [181,182]. Indeed, the role of exosomes on the spreading of α-syn pathology is gaining attention, and it was recently shown that exosomes isolated from plasma of PD patients contain higher levels of α-syn when compared to exosomes from control individuals [183,184].It is also possible that exosomal encapsulation of α-syn might confer partial protection against extracellular protein degradation mechanisms. However, the precise role of exosomes in the spreading of α-syn pathology needs to be further detailed.

Other putative mechanisms involved in cell-to-cell transfer of α-syn include endocytosis. This was confirmed in experiments using dynasore, a potent endocytosis inhibitor that dramatically reduces α-syn uptake [163]. Nevertheless, no α-syn-specific endocytic receptor has been described thus far. Alternative intercellular propagation pathways, such as transmission through axonal transport [185], or via a trans-synaptic mechanism [186], have been proposed. Importantly, the intracellular trafficking of α-syn was found to be both anterograde and retrograde [187].

Another outstanding issue in the field relates to the factor(s) initiating the transfer and spreading of α-syn, since α-syn is primarily a cytoplasmic protein of which only a small fraction appears to be secreted under physiological conditions [180]. Stress conditions, promoting disturbances of cellular proteostasis, such as impairment of lysosomal and proteasomal function, and protein misfolding, are associated with an increase of α-syn release from cells [187].

Several factors enhance α-syn secretion, including dopamine, which specifically promotes secretion of α-syn aggregates [188], or the PD-associated Leucine-rich repeat kinase 2 (LRRK2), found not only to promote α-syn release but also the cell-to-cell transmission process [189]. Importantly, mutations in the *SNCA* gene associated with familial forms of PD also increase α-syn secretion from SH-SY5Y cells [190,191].

Nevertheless, despite recent progress, it seems that we are only at the tip of the iceberg concerning the molecular underpinnings of α-syn spreading, and much more research needs to be carried out to enable a full understanding of the propagation mechanisms and the dissemination of human synucleinopathies throughout the nervous system.

### 3.4. What Species of α-Syn are Transmitted and Spread the Pathology?

An important feature of prion proteins is that they can adopt different strains, defined as structurally stable isoforms of the same polypeptide sequence displaying distinct biochemical and clinical characteristics that are associated to the development of pathology in the host organism [192]. Thus, a corollary of the prion-like hypothesis for the spreading of α-syn pathology is the possible existence of α-syn strains. Indeed, the broad pathological heterogeneity of synucleinopathies is still an intriguing phenomenon that could, perhaps, be explained by the existence of distinct α-syn strains. Although this possibility still needs to be proved, a recent study demonstrated different proteinase K digestion patterns of α-syn extracted from brains of individuals who had suffered from different synucleinopathies [175]. Although preliminary, this finding establishes the basis for additional research in larger cohorts.

α-Syn, as most amyloidogenic proteins, can be manipulated *in vitro* to generate distinct types of aggregates. This can be achieved by modulating the self-assembly conditions , by the inclusion of co-factors, such as metal ions or lipids , or due to the presence of mutations [190,193,194,195,196,197,198,199,200]. Besides structural differences, a prominent hallmark of protein strains is their seeding and self-propagation capacity. In this sense, some initial studies about the intercellular transmission of α-syn already showed that distinct oligomeric species might display distinct seeding and transmission efficiencies [201,202]. In one of the pioneering studies, variation of the ionic strength of the assembly buffer resulted in the formation of two distinct α-syn conformers that displayed distinct physico-chemical properties, self-propagation ability and toxicity [203]. Using a different number of iterative seeding cycles, another group described the generation of two recombinant α-syn species that, despite minor structural variation, exhibited striking differences in toxicity and in the cross-seeding ability over tau protein aggregation in cultured neurons. The differential tau cross-seeding efficiency of both strains was also confirmed in a mouse model of tau pathology [175]. The putative existence of α-syn strains might have a relevant impact in the spreading of the pathology, since one of the key features of a strain can be its self-propagation ability [203]. A provocative hypothesis could be that α-syn strains with a more aggressive propagation activity are the ones involved in pathology, but isolation of α-syn strains from various types of diseased brains is necessary to explore this possibility.

## 4. Interplay between α-Syn Clearance Mechanisms and Spreading

### 4.1. Lysosomal Activity and the Generation of Spreading-Competent α-Syn Species

Cellular protein homeostasis is maintained through the combined work of several pathways [204]. In some instances, it is possible for the cell to compensate for the impairment or failure of one pathway through the involvement/recruitment of another. Thus, it is understandable that the majority of data gathered thus far suggest an increase of protein release/secretion under autophagic dysfunction. In fact, it has been shown that either pharmacological or genetic impairment of autophagy lead to increased α-syn exocytosis and cell-to-cell transfer, promoting increased cellular toxicity in the cells receiving α-syn [205]. Furthermore, it was reported that secretion of α-syn oligomers via exosomes strongly depends on the stability of autophagy [181]. Exosomes originate in MVBs that originate from endosomes using a complex lipid and protein machinery, and have now become one of the most intriguing intracellular compartments due to the range of putative functions they perform in the cell [206]. Normally, MVBs transport their content to the lysosomal compartment. However, upon certain cellular stimuli, MVBs form exosomes that will later fuse with the cytoplasmic membrane. MVB membrane proteins have been described as crucial players in determining the fate of vesicles. In fact, the PD-associated protein ATP13A2 is one of those proteins, since its intracellular levels influence the fate of α-syn: lysosomal degradation or exosomal release [207,208,209]. An identical paradigm has been postulated for another MVB protein, HDAC6, which can also have a role in tau inclusion formation by impairing autophagy [141,210]. Furthermore, autophagy induction has been shown to promote fusion of MVBs with the lysosome, and inhibit exosomal release [211]. Interestingly, it was recently reported that blockade of macroautophagy promotes different secretion mechanisms depending on the α-syn molecular species. While small aggregates were secreted in Rab11a-related exosomes, larger aggregates were released in a passive method via membrane shredding [212].

**Figure 3:**
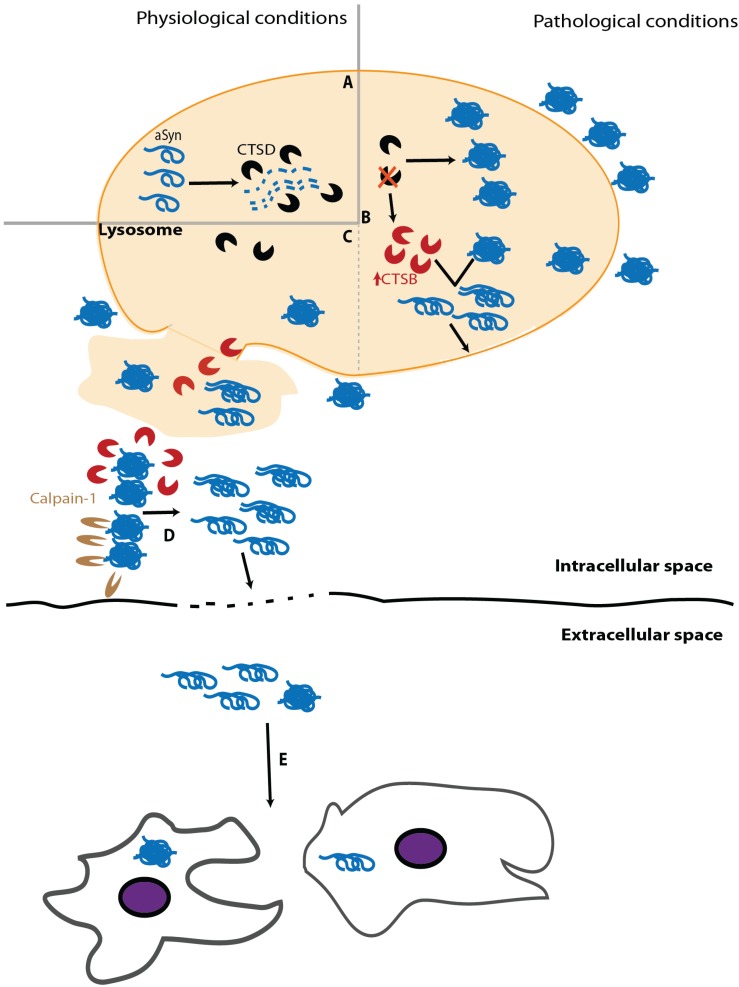
Lysosomal dysfunction can promote the release of miscleaved aggregation-prone α-syn species. (**A**) Under physiological conditions, CTSD is the main lysosomal enzyme clearing α-syn. (**B**) Deficient CTSD function promotes α-syn aggregation and lysosomal dysregulation, including the up-regulation of other enzymes such as CTSB that can convert α-syn aggregates into seeding particles able to break and escape from lysosome. (**C**) Lysosomal rupture releases the content of the organelle (e.g., lysosomal enzymes and toxic α-syn species). (**D**) These protein particles can be targeted by Calpain1, whose activity, together with that of CTSB, also generates α-syn propagating species. (**E**) α-syn toxic species can be released to the extracellular space by unknown mechanisms or by cell death derived from lysosomal rupture and be internalized by surrounding cells.

Impairment of α-syn clearance caused by lysosomal dysfunction can be due to enzymatic failure. A relevant player of this paradigm is glucocerebrosidade (GCase). This lysosomal hydrolase is associated with Gaucher’s disease, the most common lysosomal storage disease (LSD) [213]. It has been shown that GCase deficiency leads to neuroinflammation and α-syn accumulation [214] and that this depletion can also promote α-syn intercellular transmission [215]. Interestingly, α-syn can inhibit GCase activity, thereby enhancing α-syn aggregation [216]. Among lysosomal enzymes, CTSD plays a prominent role as it is the major enzyme processing α-syn in the lysosome (Figure 3A) [147]. Despite the growing number of studies devoted to study the role of CTSD in PD and other synucleinopathies, its activity in diseased brains has been poorly explored. Initial studies revealed similar activity levels of CTSD in the brain and CSF of PD and control individuals [217,218]. However, an exhaustive histochemical analysis of individual neurons in the *susbtantia nigra* of PD revealed a decrease in CSTD staining, compared to age-matched controls, particularly in cells containing α-syn inclusions [219]. Although the functional interpretation of this finding is not trivial, experiments with CTSD deficient animal models provided evidence that impairment of CTSD function impairs α-syn clearance, leading to its aggregation in neurons (Figure 3B) [149,220]. Importantly, CTSD deficiency entails other deleterious consequences, including reduction of proteasome activity, which in turn also enhances α-syn accumulation, and up-regulation of other cathepsins as a compensatory cellular mechanism [220]. Although the increased levels of other lysosomal enzymes might, in principle, alleviate α-syn aggregation, this does not seem to work and might result in a two-edged sword. In this regard, increased cathepsin B (CTSB) levels, resulting from CTSD deficiency, display a detrimental activity over α-syn aggregates, generating fragments with high seeding efficiency that eventually cause the rupture of lysosome, promoting an abrupt alteration of the whole cell homeostasis leading to cell death (Figure 3B) [221]. Considering that extracellular α-syn aggregates can, upon internalization, induce lysosome rupture [222], the putative cleavage of α-syn by CTSB would create a damaging situation where the cytoplasm would be deficient in lysosomes and burdened with α-syn seeds and CTSB molecules, which can be active in the cytosol potentially amplifying the amount of α-syn seeds (Figure 3C) [223]. The eventual cell death caused by lysosomal rupture and release of the cytoplasmic content into the extracellular space might induce a pro-inflammatory response [222], as well as the spreading of α-syn seeds that could trigger a similar process in neighboring cells, thereby propagating α-syn pathology (Figure 3C–E).

Moreover, autophagy impairment can take place at an early stage, preventing the proper generation of autophagosomes and, therefore, avoiding the confinement of the α-syn species that need to be cleared. In this case, cytoplasmic proteases come into play, particularly Calpain1, a Ca^2+^-dependent cytoplasmic enzyme that cleaves soluble α-syn in several regions, including the NAC region, thus preventing α-syn self-assembly [81]. On the contrary, Calpain1 activity on aggregated α-syn occurs mainly in the C-terminus, generating highly amyloidogenic fragments (Figure 3D) [81]. Various studies, including *in vivo* experiments, have widely demonstrated that Calpain1 is able to generate C-terminally truncated α-syn fragments that are prone to aggregation [82,224,225]. Accordingly, PD midbrain samples show increased activity of Calpain1 compared to controls [226] and Calpain1-cleaved α-syn species have been detected in LBs from the brains of individuals who had PD or DLB [224].

Mouse models of α-syn aggregation with decreased Calpain1 activity present a reduced number of α-syn inclusions and alleviated nigral degeneration and synaptic defects. In addition, Calpain1 inhibition also reduces the levels of the astrocytic marker GFAP [225,226], suggesting a possible reduction of the inflammatory response. Moreover, antibodies blocking Calpain1-induced α-syn truncation prevent the intercellular propagation of α-syn and neurodegeneration in a mouse model of PD [227]. One possibility is that this effect occurs at the extracellular space, where Calpain1 is secreted along with α-syn species [227]. Considering that α-syn accumulation is a known enhancer of Calpain1 secretion [228], one can speculate that this vicious cycle contributes to the spreading of α-syn pathology and inflammation. In agreement with this hypothesis, a relevant relationship between α-syn and Ca^2+^ has also been put forward [229]. Ca^2+^ homeostasis might be strongly affected by α-syn, as it was demonstrated that transgenic mice overexpressing α-syn present long-lasting Ca^2+^ transients [230]. Ca^2+^ dysregulation has also been reported to be caused by exogenous α-syn [231], which promotes Ca^2+^ influx and, eventually, Calpain activation. Therefore, it seems that α-syn accumulation is an enhancer of Calpain1 activity and *vice-versa*, accounting for numerous detrimental effects, including α-syn pathology and a massive dysregulation of Ca^2+^ homeostasis.

Although a comprehensive examination of these data is still necessary, this may suggest a novel interpretation of the calpain-cathepsin hypothesis that was put forward in the field of Alzheimer’s disease [232,233]—in the case of PD, the final outcome would not only be cell necrosis but also the spreading of α-syn pathology.

### 4.2. Macrosecretion and Lysosome-Mediated Exocytosis

A recent wave of discoveries brought a new meaning to the interaction between the autophagy and secretory pathways, which has been highly neglected in the PD field thus far.

On one hand, the macroautophagy machinery can regulate an unconventional (ER-to-Golgi independent) pathway of extracellular protein secretion, henceforth simply designated as macrosecretion. As previously mentioned, parts of the membranes forming the phagosome originate from the ER [234]. It is believed that the same process is at the basis of macrosecretion [235]. Although little is known about the molecular mechanisms involved, Atg5, GRASP55, and Rab8a were found to be crucial players [236]. Thus, autophagy inhibition also leads to macrosecretion blockade, possibly leading to intracellular protein accumulation [237].

**Figure 4:**
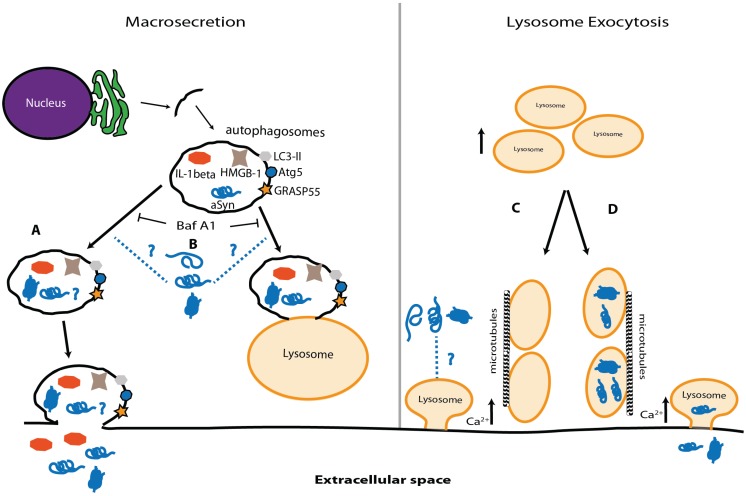
Macrosecretion and lysosome-mediated exocytosis. (**A**) Since autophagosomes can be secreted to the extracellular space, it is possible that α-syn release occurs via this pathway, both in normal conditions and upon autophagosome accumulation due to lysosome impairment; (**B**) α-syn can alter the levels of important players in macroautophagy and eventually play a role in macrosecretion; (**C**) Lysosomes can fuse with the plasma membrane and release their content, a mechanism named lysosome exocytosis. The process is similar to the one observed in neurotransmitter release, α-syn may play a role; (**D**) Alternatively, impairment of protein degradation might promote the accumulation of high molecular weight species of α-syn inside the lysosome. One can speculate that, as observed in LSDs, the lysosomes might fuse with the plasma membrane and release α-syn oligomers and aggregates into the extracellular media and start the propagation of pathology.

No research has been done so far on the role of macrosecretion in either PD or other synucleinopathies but this pathway has already been linked to the intracellular accumulation of Abeta peptide upon autophagy impairment [238]. Thus, it would be very interesting to understand (1) what cellular inputs push the system towards one of the directions; (2) if α-syn can be secreted through this pathway; (3) if α-syn can also affect macrosecretion; and (4) if inflammation in PD is associated with the release of cytokines via macrosecretion (Figure 4A,B).

Lysosome exocytosis is beneficial in LSDs [239] but its possible role in PD and other synucleinopathies has not been analysed. Lysosome exocytosis, an “unconventional secretion pathway”, shares several resemblances with synaptic vesicle release, including a Ca^2+^ dependency [240].

Lysosomal exocytosis has been associated with plasma membrane repair and immune responses [241,242]. Interestingly, it is regulated by transcription factor EB (TFEB) [239], a protein that has also been linked to the clearance of α-syn via the lysosome [243]. Thus far, little is known about α-syn in this pathway. Since it has been postulated that α-syn has an important role in neurotransmitter release [244,245], the question is whether it can also modulate lysosome exocytosis, and whether α-syn can be released to the extracellular environment via lysosome secretion upon autophagy impairment (Figure 4C,D).

### 4.3. Extracellular α-Syn Species

Among other possibilities extensively reviewed elsewhere [17,178], sources of extracellular α-syn include (i) exocytosis induced by autophagy dysfunction and (ii) passive release due to pathological conditions that eventually lead to cell death. Both possibilities have been demonstrated in different cellular and *in vivo* models, and the most plausible situation is that both take place depending on the cell type, physiological state and α-syn species. One important issue, yet unresolved, is the fate and/or the physiological role of the α-syn species that are present in the extracellular space. The clearance of extracellular α-syn species can be achieved through proteolysis by extracellular proteases or uptake by surrounding cells. The first possibility has been demonstrated in cellular models, and distinct enzymes have been found to cleave extracellular α-syn. These include neurosin [246], plasmin [247] and matrix metalloproteases [248] but the outcome of their activity on α-syn-induced toxicity is still debatable. The role of these extracellular proteases exceeds the scope of this review and readers are referred to other reviews [249].

The internalization of α-syn species by neighboring cells can be also regarded as a clearing mechanism, particularly when microglia are involved [250], although in this case, it may also entail opposing effects. As explained above, the internalization of pathological forms of α-syn can seed the pathogenic conversion of soluble α-syn in the receiving cell, and this might be one of the fundamental steps for α-syn propagation and spreading of pathology (Figure 3E) [178]. This effect has been well described in surrounding neurons where new α-syn inclusions are formed [185] and in astrocytes where α-syn can trigger aggregation and an inflammatory response [251].

### 4.4. The Putative Role of Glial Cells in the Spreading of α-Syn Pathology

PD, as other neurodegenerative disorders, is characterized by neuroinflammation in specific brain regions [252,253]. This process includes the enrolment of various types of brain cells such as microglia and astrocytes [254,255,256] and the accumulation of different molecules involved in the neuroinflammatory response [257]. However, little is known about the precise relationship between α-syn pathology and neuroinflammation, and its role in disease progression and pathology propagation [258,259].

Although α-syn is mainly a neuronal protein [260], α-syn inclusions can occur in astrocytes and oligodendrocytes, in a manner that correlates with nigral degeneration [261,262]. The analysis of PD brains showed α-syn inclusions in astrocytes with a particular distribution pattern suggesting that misfolded or abnormal α-syn molecules may escape from damaged terminal axons and then be integrated by neighboring astrocytes [263], in agreement with the current hypothesis of prion-like spreading of α-syn pathology [251]. Likewise, α-syn can be taken up by oligodendrocytes [264] and induce the formation of cytoplasmic inclusions [265]. In addition to inclusion formation, α-syn accumulation might be a relevant inducer of the local inflammatory response, as suggested by the addition of exogenous α-syn in cultured astrocytes and by the analysis of MSA brains, where astrocyte activation was greater in the vicinity of α-syn inclusions [265].

Although microglia activation is also evident in PD [256,266], its precise role in pathology progression is yet unknown. On one side, extracellular α-syn species are internalized by microglia, activating an inflammatory response and contributing to disease spreading [267]. On the other side, this phagocytic-like activity of microglia, internalizing α-syn, might also be neuroprotective, contributing to the clearance of extracellular α-syn aggregates [250] and, thereby, stopping α-syn propagation. This latter property has been successfully exploited to design a therapeutic strategy based on the microglial clearance of antibody-targeted extracellular α-syn [268]. Microglial phagocytosis of extracellular α-syn can be mediated by Toll-Like Receptors (TLRs) [269]. Whereas ablation of TLR4 in a mouse model of multiple system atrophy resulted in the accumulation of α-syn and neuronal loss [270], other studies revealed that TLR2 receptors seem to be specific for oligomeric species of α-syn [271].Indeed, α-syn conformation of appears to be crucial and, since monomeric extracellular α-syn enhances microglial phagocytosis, aggregated species inhibit this activity [272]. While these observations are still hard to reconcile, one can speculate that perhaps aggregated species of α-syn might, at some point, hinder the intracellular clearance systems, therefore causing glial cells to also contribute to the spreading process.

## 5. Conclusions

The activity of protein clearance mechanisms depends on the physiological state of the cell and its surrounding environment. They play a crucial role in clearing misfolded protein species that may appear and have deleterious consequences for the cell. The kinetics of protein aggregation, following a sigmoidal process, affords biological systems an opportunity to interfere with protein aggregation in the early rate-limiting step, thus, protecting the cell from potentially dangerous, aggregation-prone species. However, for reasons that are currently still unclear, some proteins may escape the cellular quality control systems, misfold, and aggregate, leading to a variety of detrimental consequences, including cell death. In some instances, cellular efforts to counteract the protein aggregation process, through the activation of protein degradation pathways, might become two-edged swords, enhancing protein aggregation and the generation of species that can be transmitted to neighboring cells.

Although the majority of studies performed thus far have focused on the role of autophagy-lysosomal pathway on α-syn propagation, it will also be important to assess the role of the UPS in this process, since the impairment of this pathway might precipitate a cascade of events culminating in increased extracellular release and, thereby, spreading of pathology. Ultimately, we posit that the comprehension of the synergistic and compensatory mechanisms of these pathways might lead to the identification of novel targets and strategies for modulating the spreading of pathology in PD and other synucleinopathies.

## References

[1] Marti M.J., Tolosa E., Campdelacreu J. (2003). Clinical overview of the synucleinopathies. Mov. Disord..

[2] Chaudhuri K.R., Naidu Y. (2008). Early Parkinson’s disease and non-motor issues. J. Neurol..

[3] Harding A.J., Stimson E., Henderson J.M., Halliday G.M. (2002). Clinical correlates of selective pathology in the amygdala of patients with Parkinson’s disease. Brain.

[4] Braak H., del Tredici K., Rub U., de Vos R.A., Jansen Steur E.N., Braak E. (2003). Staging of brain pathology related to sporadic Parkinson’s disease. Neurobiol. Aging.

[5] Baba T., Kikuchi A., Hirayama K., Nishio Y., Hosokai Y., Kanno S., Hasegawa T., Sugeno N., Konno M., Suzuki K. (2012). Severe olfactory dysfunction is a prodromal symptom of dementia associated with Parkinson’s disease: A 3 year longitudinal study. Brain.

[6] Moessnang C., Frank G., Bogdahn U., Winkler J., Greenlee M.W., Klucken J. (2011). Altered activation patterns within the olfactory network in Parkinson’s disease. Cereb. Cortex.

[7] Wang J., You H., Liu J.F., Ni D.F., Zhang Z.X., Guan J. (2011). Association of olfactory bulb volume and olfactory sulcus depth with olfactory function in patients with Parkinson disease. Am. J. Neuroradiol..

[8] Ansari K.A., Johnson A. (1975). Olfactory function in patients with Parkinson’s disease. J. Chronic Dis..

[9] Simuni T., Sethi K. (2008). Nonmotor manifestations of Parkinson’s disease. Ann. Neurol..

[10] Park A., Stacy M. (2009). Non-motor symptoms in Parkinson’s disease. J. Neurol..

[11] Gallagher D.A., Lees A.J., Schrag A. (2010). What are the most important nonmotor symptoms in patients with Parkinson’s disease and are we missing them?. Mov. Disord..

[12] Olanow C.W., Goetz C.G., Kordower J.H., Stoessl A.J., Sossi V., Brin M.F., Shannon K.M., Nauert G.M., Perl D.P., Godbold J. (2003). A double-blind controlled trial of bilateral fetal nigral transplantation in Parkinson’s disease. Ann. Neurol..

[13] Lindvall O., Sawle G., Widner H., Rothwell J.C., Bjorklund A., Brooks D., Brundin P., Frackowiak R., Marsden C.D., Odin P. (1994). Evidence for long-term survival and function of dopaminergic grafts in progressive Parkinson’s disease. Ann. Neurol..

[14] Kordower J.H., Freeman T.B., Snow B.J., Vingerhoets F.J., Mufson E.J., Sanberg P.R., Hauser R.A., Smith D.A., Nauert G.M., Perl D.P. (1995). Neuropathological evidence of graft survival and striatal reinnervation after the transplantation of fetal mesencephalic tissue in a patient with Parkinson’s disease. N. Engl. J. Med..

[15] Li J.Y., Englund E., Holton J.L., Soulet D., Hagell P., Lees A.J., Lashley T., Quinn N.P., Rehncrona S., Bjorklund A. (2008). Lewy bodies in grafted neurons in subjects with Parkinson’s disease suggest host-to-graft disease propagation. Nat. Med..

[16] Kordower J.H., Chu Y., Hauser R.A., Freeman T.B., Olanow C.W. (2008). Lewy body-like pathology in long-term embryonic nigral transplants in Parkinson’s disease. Nat. Med..

[17] Wales P., Pinho R., Lazaro D.F., Outeiro T.F. (2013). Limelight on alpha-synuclein: Pathological and mechanistic implications in neurodegeneration. J. Parkinsons Dis..

[18] Polymeropoulos M.H., Lavedan C., Leroy E., Ide S.E., Dehejia A., Dutra A., Pike B., Root H., Rubenstein J., Boyer R. (1997). Mutation in the alpha-synuclein gene identified in families with Parkinson’s disease. Science.

[19] Kruger R., Kuhn W., Muller T., Woitalla D., Graeber M., Kosel S., Przuntek H., Epplen J.T., Schols L., Riess O. (1998). Ala30pro mutation in the gene encoding alpha-synuclein in Parkinson’s disease. Nat. Genet..

[20] Zarranz J.J., Alegre J., Gomez-Esteban J.C., Lezcano E., Ros R., Ampuero I., Vidal L., Hoenicka J., Rodriguez O., Atares B. (2004). The new mutation, E46K, of alpha-synuclein causes parkinson and Lewy body dementia. Ann. Neurol..

[21] Appel-Cresswell S., Vilarino-Guell C., Encarnacion M., Sherman H., Yu I., Shah B., Weir D., Thompson C., Szu-Tu C., Trinh J. (2013). Alpha-synuclein p.H50Q, a novel pathogenic mutation for Parkinson’s disease. Mov. Disord..

[22] Lesage S., Anheim M., Letournel F., Bousset L., Honore A., Rozas N., Pieri L., Madiona K., Durr A., Melki R. (2013). G51D alpha-synuclein mutation causes a novel parkinsonian-pyramidal syndrome. Ann. Neurol..

[23] Pasanen P., Myllykangas L., Siitonen M., Raunio A., Kaakkola S., Lyytinen J., Tienari P.J., Poyhonen M., Paetau A. (2014). A novel alpha-synuclein mutation A53E associated with atypical multiple system atrophy and Parkinson’s disease-type pathology. Neurobiol. Aging.

[24] Singleton A.B., Farrer M., Johnson J., Singleton A., Hague S., Kachergus J., Hulihan M., Peuralinna T., Dutra A., Nussbaum R. (2003). Alpha-synuclein locus triplication causes Parkinson’s disease. Science.

[25] Chartier-Harlin M.C., Kachergus J., Roumier C., Mouroux V., Douay X., Lincoln S., Levecque C., Larvor L., Andrieux J., Hulihan M. (2004). Alpha-synuclein locus duplication as a cause of familial Parkinson’s disease. Lancet.

[26] Satake W., Nakabayashi Y., Mizuta I., Hirota Y., Ito C., Kubo M., Kawaguchi T., Tsunoda T., Watanabe M., Takeda A. (2009). Genome-wide association study identifies common variants at four loci as genetic risk factors for Parkinson’s disease. Nat. Genet..

[27] Simon-Sanchez J., Schulte C., Bras J.M., Sharma M., Gibbs J.R., Berg D., Paisan-Ruiz C., Lichtner P., Scholz S.W., Hernandez D.G. (2009). Genome-wide association study reveals genetic risk underlying Parkinson’s disease. Nat. Genet..

[28] Edwards T.L., Scott W.K., Almonte C., Burt A., Powell E.H., Beecham G.W., Wang L., Zuchner S., Konidari I., Wang G. (2010). Genome-wide association study confirms snps in SNCA and the mapt region as common risk factors for Parkinson disease. Ann. Hum. Genet..

[29] Nalls M.A., Pankratz N., Lill C.M., Do C.B., Hernandez D.G., Saad M., DeStefano A.L., Kara E., Bras J., Sharma M. (2014). Large-scale meta-analysis of genome-wide association data identifies six new risk loci for Parkinson’s disease. Nat. Genet..

[30] Maroteaux L., Campanelli J.T., Scheller R.H. (1988). Synuclein: A neuron-specific protein localized to the nucleus and presynaptic nerve terminal. J. Neurosci..

[31] Surewicz W.K., Epand R.M., Pownall H.J., Hui S.W. (1986). Human apolipoprotein A–I forms thermally stable complexes with anionic but not with zwitterionic phospholipids. J. Biol. Chem..

[32] Vamvaca K., Volles M.J., Lansbury P.T. (2009). The first N-terminal amino acids of alpha-synuclein are essential for alpha-helical structure formation *in vitro* and membrane binding in yeast. J. Mol. Biol..

[33] Bartels T., Ahlstrom L.S., Leftin A., Kamp F., Haass C., Brown M.F., Beyer K. (2010). The N-terminus of the intrinsically disordered protein alpha-synuclein triggers membrane binding and helix folding. Biophys. J..

[34] Giasson B.I., Murray I.V., Trojanowski J.Q., Lee V.M. (2001). A hydrophobic stretch of 12 amino acid residues in the middle of alpha-synuclein is essential for filament assembly. J. Biol. Chem..

[35] Izawa Y., Tateno H., Kameda H., Hirakawa K., Hato K., Yagi H., Hongo K., Mizobata T., Kawata Y. (2012). Role of C-terminal negative charges and tyrosine residues in fibril formation of alpha-synuclein. Brain Behav..

[36] Nielsen M.S., Vorum H., Lindersson E., Jensen P.H. (2001). Ca^2+^ binding to alpha-synuclein regulates ligand binding and oligomerization. J. Biol. Chem..

[37] Lowe R., Pountney D.L., Jensen P.H., Gai W.P., Voelcker N.H. (2004). Calcium(II) selectively induces alpha-synuclein annular oligomers via interaction with the C-terminal domain. Protein Sci..

[38] Souza J.M., Giasson B.I., Lee V.M., Ischiropoulos H. (2000). Chaperone-like activity of synucleins. FEBS Lett..

[39] Bartels T., Choi J.G., Selkoe D.J. (2011). Alpha-synuclein occurs physiologically as a helically folded tetramer that resists aggregation. Nature.

[40] Wang W., Perovic I., Chittuluru J., Kaganovich A., Nguyen L.T., Liao J., Auclair J.R., Johnson D., Landeru A., Simorellis A.K. (2011). A soluble alpha-synuclein construct forms a dynamic tetramer. Proc. Natl. Acad. Sci. USA.

[41] Dettmer U., Newman A.J., Luth E.S., Bartels T., Selkoe D. (2013). *In vivo* cross-linking reveals principally oligomeric forms of alpha-synuclein and beta-synuclein in neurons and non-neural cells. J. Biol. Chem..

[42] Fauvet B., Mbefo M.K., Fares M.B., Desobry C., Michael S., Ardah M.T., Tsika E., Coune P., Prudent M., Lion N. (2012). Alpha-synuclein in central nervous system and from erythrocytes, mammalian cells, and escherichia coli exists predominantly as disordered monomer. J. Biol. Chem..

[43] Outeiro T.F., Putcha P., Tetzlaff J.E., Spoelgen R., Koker M., Carvalho F., Hyman B.T., McLean P.J. (2008). Formation of toxic oligomeric alpha-synuclein species in living cells. PLOS ONE.

[44] Winner B., Jappelli R., Maji S.K., Desplats P.A., Boyer L., Aigner S., Hetzer C., Loher T., Vilar M., Campioni S. (2011). *In vivo* demonstration that alpha-synuclein oligomers are toxic. Proc. Natl. Acad. Sci. USA.

[45] Karpinar D.P., Balija M.B., Kugler S., Opazo F., Rezaei-Ghaleh N., Wender N., Kim H.Y., Taschenberger G., Falkenburger B.H., Heise H. (2009). Pre-fibrillar alpha-synuclein variants with impaired beta-structure increase neurotoxicity in Parkinson’s disease models. MBO J..

[46] El-Agnaf O.M., Jakes R., Curran M.D., Middleton D., Ingenito R., Bianchi E., Pessi A., Neill D., Wallace A. (1998). Aggregates from mutant and wild-type alpha-synuclein proteins and NAC peptide induce apoptotic cell death in human neuroblastoma cells by formation of beta-sheet and amyloid-like filaments. FEBS Lett..

[47] Tanik S.A., Schultheiss C.E., Volpicelli-Daley L.A., Brunden K.R., Lee V.M. (2013). Lewy body-like alpha-synuclein aggregates resist degradation and impair macroautophagy. J. Biol. Chem..

[48] Anderson J.P., Walker D.E., Goldstein J.M., de Laat R., Banducci K., Caccavello R.J., Barbour R., Huang J., Kling K., Lee M. (2006). Phosphorylation of Ser-129 is the dominant pathological modification of alpha-synuclein in familial and sporadic Lewy body disease. J. Biol. Chem..

[49] Tofaris G.K., Razzaq A., Ghetti B., Lilley K.S., Spillantini M.G. (2003). Ubiquitination of alpha-synuclein in lewy bodies is a pathological event not associated with impairment of proteasome function. J. Biol. Chem..

[50] Rott R., Szargel R., Haskin J., Shani V., Shainskaya A., Manov I., Liani E., Avraham E., Engelender S. (2008). Monoubiquitylation of alpha-synuclein by seven in absentia homolog (SIAH) promotes its aggregation in dopaminergic cells. J. Biol. Chem..

[51] Shin Y., Klucken J., Patterson C., Hyman B.T., McLean P.J. (2005). The co-chaperone carboxyl terminus of Hsp70-interacting protein (CHIP) mediates alpha-synuclein degradation decisions between proteasomal and lysosomal pathways. J. Biol. Chem..

[52] Krumova P., Meulmeester E., Garrido M., Tirard M., Hsiao H.H., Bossis G., Urlaub H., Zweckstetter M., Kugler S., Melchior F. (2011). Sumoylation inhibits alpha-synuclein aggregation and toxicity. J. Cell Biol..

[53] Kim Y.M., Jang W.H., Quezado M.M., Oh Y., Chung K.C., Junn E., Mouradian M.M. (2011). Proteasome inhibition induces alpha-synuclein sumoylation and aggregate formation. J. Neurol. Sci..

[54] Shahpasandzadeh H., Popova B., Kleinknecht A., Fraser P.E., Outeiro T.F., Braus G.H. (2014). Interplay between sumoylation and phosphorylation for protection against alpha-synuclein inclusions. J. Biol. Chem..

[55] Dikiy I., Eliezer D. (2014). N-terminal acetylation stabilizes N-terminal helicity in lipid- and micelle-bound alpha-synuclein and increases its affinity for physiological membranes. J. Biol. Chem..

[56] Maltsev A.S., Ying J., Bax A. (2012). Impact of N-terminal acetylation of alpha-synuclein on its random coil and lipid binding properties. Biochemistry.

[57] Bartels T., Kim N.C., Luth E.S., Selkoe D.J. (2014). N-alpha-acetylation of alpha-synuclein increases its helical folding propensity, gm1 binding specificity and resistance to aggregation. PLOS ONE.

[58] Okochi M., Walter J., Koyama A., Nakajo S., Baba M., Iwatsubo T., Meijer L., Kahle P.J., Haass C. (2000). Constitutive phosphorylation of the Parkinson’s disease associated alpha-synuclein. J. Biol. Chem..

[59] Fujiwara H., Hasegawa M., Dohmae N., Kawashima A., Masliah E., Goldberg M.S., Shen J., Takio K., Iwatsubo T. (2002). Alpha-synuclein is phosphorylated in synucleinopathy lesions. Nat. Cell Biol..

[60] Pronin A.N., Morris A.J., Surguchov A., Benovic J.L. (2000). Synucleins are a novel class of substrates for G protein-coupled receptor kinases. J. Biol. Chem..

[61] Arawaka S., Wada M., Goto S., Karube H., Sakamoto M., Ren C.H., Koyama S., Nagasawa H., Kimura H., Kawanami T. (2006). The role of G-protein-coupled receptor kinase 5 in pathogenesis of sporadic Parkinson’s disease. J. Neurosci..

[62] Inglis K.J., Chereau D., Brigham E.F., Chiou S.S., Schobel S., Frigon N.L., Yu M., Caccavello R.J., Nelson S., Motter R. (2009). Polo-like kinase 2 (PLK2) phosphorylates alpha-synuclein at serine 129 in central nervous system. J. Biol. Chem..

[63] Mbefo M.K., Paleologou K.E., Boucharaba A., Oueslati A., Schell H., Fournier M., Olschewski D., Yin G., Zweckstetter M., Masliah E. (2010). Phosphorylation of synucleins by members of the polo-like kinase family. J. Biol. Chem..

[64] Ishii A., Nonaka T., Taniguchi S., Saito T., Arai T., Mann D., Iwatsubo T., Hisanaga S., Goedert M., Hasegawa M. (2007). Casein kinase 2 is the major enzyme in brain that phosphorylates ser129 of human alpha-synuclein: Implication for alpha-synucleinopathies. FEBS Lett..

[65] Basso E., Antas P., Marijanovic Z., Goncalves S., Tenreiro S., Outeiro T.F. (2013). PLK2 modulates alpha-synuclein aggregation in yeast and mammalian cells. Mol. Neurobiol..

[66] Ryu M.Y., Kim D.W., Arima K., Mouradian M.M., Kim S.U., Lee G. (2008). Localization of CKII beta subunits in lewy bodies of Parkinson’s disease. J. Neurol. Sci..

[67] Chen L., Feany M.B. (2005). Alpha-synuclein phosphorylation controls neurotoxicity and inclusion formation in a drosophila model of Parkinson disease. Nat. Neurosci..

[68] Tenreiro S., Reimao-Pinto M.M., Antas P., Rino J., Wawrzycka D., Macedo D., Rosado-Ramos R., Amen T., Waiss M., Magalhaes F. (2014). Phosphorylation modulates clearance of alpha-synuclein inclusions in a yeast model of Parkinson’s disease. PLOS Genet..

[69] Paleologou K.E., Oueslati A., Shakked G., Rospigliosi C.C., Kim H.Y., Lamberto G.R., Fernandez C.O., Schmid A., Chegini F., Gai W.P. (2010). Phosphorylation at S87 is enhanced in synucleinopathies, inhibits alpha-synuclein oligomerization, and influences synuclein-membrane interactions. J. Neurosci..

[70] Oueslati A., Fournier M., Lashuel H.A. (2010). Role of post-translational modifications in modulating the structure, function and toxicity of alpha-synuclein: Implications for Parkinson’s disease pathogenesis and therapies. Prog. Brain Res..

[71] Sato H., Kato T., Arawaka S. (2013). The role of Ser129 phosphorylation of alpha-synuclein in neurodegeneration of Parkinson’s disease: A review of *in vivo* models. Rev. Neurosci..

[72] Tenreiro S., Eckermann K., Outeiro T.F. (2014). Protein phosphorylation in neurodegeneration: Friend or foe?. Front. Mol. Neurosci..

[73] Giasson B.I., Duda J.E., Murray I.V., Chen Q., Souza J.M., Hurtig H.I., Ischiropoulos H., Trojanowski J.Q., Lee V.M. (2000). Oxidative damage linked to neurodegeneration by selective alpha-synuclein nitration in synucleinopathy lesions. Science.

[74] Liu Y., Qiang M., Wei Y., He R. (2011). A novel molecular mechanism for nitrated α-synuclein-induced cell death. J. Mol. Cell Biol..

[75] Yu Z., Xu X., Xiang Z., Zhou J., Zhang Z., Hu C., He C. (2010). Nitrated alpha-synuclein induces the loss of dopaminergic neurons in the substantia nigra of rats. PLOS ONE.

[76] Hodara R., Norris E.H., Giasson B.I., Mishizen-Eberz A.J., Lynch D.R., Lee V.M., Ischiropoulos H. (2004). Functional consequences of alpha-synuclein tyrosine nitration: Diminished binding to lipid vesicles and increased fibril formation. J. Biol. Chem..

[77] Li W., West N., Colla E., Pletnikova O., Troncoso J.C., Marsh L., Dawson T.M., Jakala P., Hartmann T., Price D.L. (2005). Aggregation promoting C-terminal truncation of alpha-synuclein is a normal cellular process and is enhanced by the familial Parkinson’s disease-linked mutations. Proc. Natl. Acad. Sci. USA.

[78] Baba M., Nakajo S., Tu P.H., Tomita T., Nakaya K., Lee V.M., Trojanowski J.Q., Iwatsubo T. (1998). Aggregation of alpha-synuclein in lewy bodies of sporadic Parkinson’s disease and dementia with lewy bodies. Am. J. Pathol..

[79] Murray I.V., Giasson B.I., Quinn S.M., Koppaka V., Axelsen P.H., Ischiropoulos H., Trojanowski J.Q., Lee V.M. (2003). Role of alpha-synuclein carboxy-terminus on fibril formation *in vitro*. Biochemistry.

[80] Ulusoy A., Febbraro F., Jensen P.H., Kirik D., Romero-Ramos M. (2010). Co-expression of C-terminal truncated alpha-synuclein enhances full-length alpha-synuclein-induced pathology. Eur. J. Neurosci..

[81] Mishizen-Eberz A.J., Guttmann R.P., Giasson B.I., Day G.A., Hodara R., Ischiropoulos H., Lee V.M., Trojanowski J.Q., Lynch D.R. (2003). Distinct cleavage patterns of normal and pathologic forms of alpha-synuclein by calpain I *in vitro*. J. Neurochem..

[82] Mishizen-Eberz A.J., Norris E.H., Giasson B.I., Hodara R., Ischiropoulos H., Lee V.M., Trojanowski J.Q., Lynch D.R. (2005). Cleavage of alpha-synuclein by calpain: Potential role in degradation of fibrillized and nitrated species of alpha-synuclein. Biochemistry.

[83] Iwata A., Maruyama M., Akagi T., Hashikawa T., Kanazawa I., Tsuji S., Nukina N. (2003). Alpha-synuclein degradation by serine protease neurosin: Implication for pathogenesis of synucleinopathies. Hum. Mol. Genet..

[84] Kasai T., Tokuda T., Yamaguchi N., Watanabe Y., Kametani F., Nakagawa M., Mizuno T. (2008). Cleavage of normal and pathological forms of alpha-synuclein by neurosin *in vitro*. Neurosci. Lett..

[85] Cuervo A., Stefanis L., Fredenburg R., Lansbury P. (2004). Impaired degradation of mutant α-synuclein by chaperone-mediated autophagy. Science.

[86] Liu C.W., Corboy M.J., DeMartino G.N., Thomas P.J. (2003). Endoproteolytic activity of the proteasome. Science.

[87] Yang F., Yang Y.P., Mao C.J., Liu L., Zheng H.F., Hu L.F., Liu C.F. (2013). Crosstalk between the proteasome system and autophagy in the clearance of alpha-synuclein. Acta Pharmacol. Sin..

[88] Lee H.J., Khoshaghideh F., Patel S., Lee S.J. (2004). Clearance of alpha-synuclein oligomeric intermediates via the lysosomal degradation pathway. J. Neurosci..

[89] Mizushima N., Klionsky D.J. (2007). Protein turnover via autophagy: Implications for metabolism. Annu. Rev. Nutr..

[90] Dice J.F. (1990). Peptide sequences that target cytosolic proteins for lysosomal proteolysis. Trends Biochem. Sci..

[91] Kiffin R., Christian C., Knecht E., Cuervo A.M. (2004). Activation of chaperone-mediated autophagy during oxidative stress. Mol. Biol. Cell.

[92] Chiang H.L., Terlecky S.R., Plant C.P., Dice J.F. (1989). A role for a 70-kilodalton heat shock protein in lysosomal degradation of intracellular proteins. Science.

[93] Eskelinen E.L., Cuervo A.M., Taylor M.R., Nishino I., Blum J.S., Dice J.F., Sandoval I.V., Lippincott-Schwartz J., August J.T., Saftig P. (2005). Unifying nomenclature for the isoforms of the lysosomal membrane protein LAMP-2. Traffic.

[94] Cuervo A.M., Dice J.F. (1996). A receptor for the selective uptake and degradation of proteins by lysosomes. Science.

[95] Agarraberes F.A., Terlecky S.R., Dice J.F. (1997). An intralysosomal Hsp70 is required for a selective pathway of lysosomal protein degradation. J. Cell Biol..

[96] Vogiatzi T., Xilouri M., Vekrellis K., Stefanis L. (2008). Wild type alpha-synuclein is degraded by chaperone-mediated autophagy and macroautophagy in neuronal cells. J. Biol. Chem..

[97] Mak S.K., McCormack A.L., Manning-Bog A.B., Cuervo A.M., di Monte D.A. (2010). Lysosomal degradation of alpha-synuclein *in vivo*. J. Biol. Chem..

[98] Martinez-Vicente M., Talloczy Z. (2008). Dopamine-modified α-synuclein blocks chaperone-mediated autophagy. J. Clin. Invest..

[99] Xilouri M., Vogiatzi T., Vekrellis K., Park D., Stefanis L. (2009). Abberant alpha-synuclein confers toxicity to neurons in part through inhibition of chaperone-mediated autophagy. PLOS ONE.

[100] Xilouri M., Brekk O.R., Landeck N., Pitychoutis P.M., Papasilekas T., Papadopoulou-Daifoti Z., Kirik D., Stefanis L. (2013). Boosting chaperone-mediated autophagy *in vivo* mitigates alpha-synuclein-induced neurodegeneration. Brain.

[101] Cuervo A.M., Dice J.F. (2000). Age-related decline in chaperone-mediated autophagy. J. Biol. Chem..

[102] Alvarez-Erviti L., Rodriguez-Oroz M.C., Cooper J.M., Caballero C., Ferrer I., Obeso J.A., Schapira A.H. (2010). Chaperone-mediated autophagy markers in Parkinson disease brains. Arch. Neurol..

[103] Eskelinen E.L., Schmidt C.K., Neu S., Willenborg M., Fuertes G., Salvador N., Tanaka Y., Lullmann-Rauch R., Hartmann D., Heeren J. (2004). Disturbed cholesterol traffic but normal proteolytic function in LAMP-1/LAMP-2 double-deficient fibroblasts. Mol. Biol. Cell.

[104] Kim J., Inoue K., Ishii J., Vanti W.B., Voronov S.V., Murchison E., Hannon G., Abeliovich A. (2007). A microRNA feedback circuit in midbrain dopamine neurons. Science.

[105] Li G., Yang H., Zhu D., Huang H., Liu G., Lun P. (2014). Targeted suppression of chaperone-mediated autophagy by miR-320A promotes alpha-synuclein aggregation. Int. J. Mol. Sci..

[106] Alvarez-Erviti L., Seow Y., Schapira A.H., Rodriguez-Oroz M.C., Obeso J.A., Cooper J.M. (2013). Influence of microRNA deregulation on chaperone-mediated autophagy and alpha-synuclein pathology in Parkinson’s disease. Cell Death Dis..

[107] Bjersing J.L., Bokarewa M.I., Mannerkorpi K. (2015). Profile of circulating microRNAs in fibromyalgia and their relation to symptom severity: An exploratory study. Rheumatol. Int..

[108] Kubiczkova-Besse L., Sedlarikova L., Kryukov F., Nekvindova J., Radova L., Almasi M., Pelcova J., Minarik J., Pika T., Pikalova Z. (2015). Combination of serum microRNA-320A and microRNA-320B as a marker for waldenstrom macroglobulinemia. Am. J. Hematol..

[109] Sheng M., Zhong Y., Chen Y., Du J., Ju X., Zhao C., Zhang G., Zhang L., Liu K., Yang N. (2014). Hsa-miR-1246, Hsa-miR-320a and Hsa-miR-196B-5P inhibitors can reduce the cytotoxicity of ebola virus glycoprotein *in vitro*. Sci. China Life Sci..

[110] Noda T., Suzuki K., Ohsumi Y. (2002). Yeast autophagosomes: *De novo* formation of a membrane structure. Trends Cell Biol..

[111] Kraft C., Martens S. (2012). Mechanisms and regulation of autophagosome formation. Curr. Opin. Cell Biol..

[112] Reggiori F., Tucker K.A., Stromhaug P.E., Klionsky D.J. (2004). The Atg1-Atg13 complex regulates Atg9 and Atg23 retrieval transport from the pre-autophagosomal structure. Dev. Cell.

[113] Reggiori F., Shintani T., Nair U., Klionsky D.J. (2005). Atg9 cycles between mitochondria and the pre-autophagosomal structure in yeasts. Autophagy.

[114] Orsi A., Razi M., Dooley H.C., Robinson D., Weston A.E., Collinson L.M., Tooze S.A. (2012). Dynamic and transient interactions of Atg9 with autophagosomes, but not membrane integration, are required for autophagy. Mol. Biol. Cell.

[115] Young A.R., Chan E.Y., Hu X.W., Kochl R., Crawshaw S.G., High S., Hailey D.W., Lippincott-Schwartz J., Tooze S.A. (2006). Starvation and ULK1-dependent cycling of mammalian Atg9 between the TGN and endosomes. J. Cell Sci..

[116] Cardenas M.E., Cutler N.S., Lorenz M.C., di Como C.J., Heitman J. (1999). The TOR signaling cascade regulates gene expression in response to nutrients. Genes Dev..

[117] Zeng X., Overmeyer J.H., Maltese W.A. (2006). Functional specificity of the mammalian Beclin-Vps34 PI 3-kinase complex in macroautophagy *versus* endocytosis and lysosomal enzyme trafficking. J. Cell Sci..

[118] Thumm M., Egner R., Koch B., Schlumpberger M., Straub M., Veenhuis M., Wolf D.H. (1994). Isolation of autophagocytosis mutants of saccharomyces cerevisiae. FEBS Lett..

[119] Tsukada M., Ohsumi Y. (1993). Isolation and characterization of autophagy-defective mutants of saccharomyces cerevisiae. FEBS Lett..

[120] Klionsky D.J., Cregg J.M., Dunn W.A., Emr S.D., Sakai Y., Sandoval I.V., Sibirny A., Subramani S., Thumm M., Veenhuis M. (2003). A unified nomenclature for yeast autophagy-related genes. Dev. Cell.

[121] Mizushima N., Noda T., Yoshimori T., Tanaka Y., Ishii T., George M.D., Klionsky D.J., Ohsumi M., Ohsumi Y. (1998). A protein conjugation system essential for autophagy. Nature.

[122] Tanida I., Tanida-Miyake E., Ueno T., Kominami E. (2001). The human homolog of saccharomyces cerevisiae Apg7p is a protein-activating enzyme for multiple substrates including human Apg12p, GATE-16, GABARAP, and MAP-LC3. J. Biol. Chem..

[123] Mizushima N., Noda T., Ohsumi Y. (1999). Apg16p is required for the function of the Apg12p-Apg5p conjugate in the yeast autophagy pathway. EMBO J..

[124] Romanov J., Walczak M., Ibiricu I., Schuchner S., Ogris E., Kraft C., Martens S. (2012). Mechanism and functions of membrane binding by the Atg5-Atg12/Atg16 complex during autophagosome formation. EMBO J..

[125] Tanida I., Sou Y.S., Ezaki J., Minematsu-Ikeguchi N., Ueno T., Kominami E. (2004). HsAtg4b/Hsapg4b/autophagin-1 cleaves the carboxyl termini of three human Atg8 homologues and delipidates microtubule-associated protein light chain 3- and GABAA receptor-associated protein-phospholipid conjugates. J. Biol. Chem..

[126] Kabeya Y., Mizushima N., Ueno T., Yamamoto A., Kirisako T., Noda T., Kominami E., Ohsumi Y., Yoshimori T. (2000). LC3, a mammalian homologue of yeast Apg8p, is localized in autophagosome membranes after processing. EMBO J..

[127] Tanida I., Tanida-Miyake E., Komatsu M., Ueno T., Kominami E. (2002). Human Apg3p/Aut1p homologue is an authentic E2 enzyme for multiple substrates, GATE-16, GABARAP, and MAP-LC3, and facilitates the conjugation of Hapg12p to Hapg5p. J. Biol. Chem..

[128] Otomo C., Metlagel Z., Takaesu G., Otomo T. (2013). Structure of the human Atg12~Atg5 conjugate required for LC3 lipidation in autophagy. Nat. Struct. Mol. Biol..

[129] Fujita N., Itoh T., Omori H., Fukuda M., Noda T., Yoshimori T. (2008). The Atg16l complex specifies the site of LC3 lipidation for membrane biogenesis in autophagy. Mol. Biol. Cell.

[130] Olzmann J.A., Li L., Chudaev M.V., Chen J., Perez F.A., Palmiter R.D., Chin L.S. (2007). Parkin-mediated K63-linked polyubiquitination targets misfolded DJ-1 to aggresomes via binding to HDAC6. J. Cell Biol..

[131] Kawaguchi Y., Kovacs J.J., McLaurin A., Vance J.M., Ito A., Yao T.P. (2003). The deacetylase HDAC6 regulates aggresome formation and cell viability in response to misfolded protein stress. Cell.

[132] Lamark T., Perander M., Outzen H., Kristiansen K., Overvatn A., Michaelsen E., Bjorkoy G., Johansen T. (2003). Interaction codes within the family of mammalian Phox and Bem1p domain-containing proteins. J. Biol. Chem..

[133] Noda N.N., Kumeta H., Nakatogawa H., Satoo K., Adachi W., Ishii J., Fujioka Y., Ohsumi Y., Inagaki F. (2008). Structural basis of target recognition by Atg8/LC3 during selective autophagy. Genes Cells Devoted Mol. Cell. Mech..

[134] Pankiv S., Clausen T.H., Lamark T., Brech A., Bruun J.A., Outzen H., Overvatn A., Bjorkoy G., Johansen T. (2007). P62/SQSTM1 binds directly to Atg8/LC3 to facilitate degradation of ubiquitinated protein aggregates by autophagy. J. Biol. Chem..

[135] Kirkin V., Lamark T., Sou Y.S., Bjorkoy G., Nunn J.L., Bruun J.A., Shvets E., McEwan D.G., Clausen T.H., Wild P. (2009). A role for NBR1 in autophagosomal degradation of ubiquitinated substrates. Mol. Cell.

[136] Tan J.M., Wong E.S., Kirkpatrick D.S., Pletnikova O., Ko H.S., Tay S.P., Ho M.W., Troncoso J., Gygi S.P., Lee M.K. (2008). Lysine 63-linked ubiquitination promotes the formation and autophagic clearance of protein inclusions associated with neurodegenerative diseases. Hum. Mol. Genet..

[137] Seibenhener M.L., Babu J.R., Geetha T., Wong H.C., Krishna N.R., Wooten M.W. (2004). Sequestosome 1/p62 is a polyubiquitin chain binding protein involved in ubiquitin proteasome degradation. Mol. Cell. Biol..

[138] Zhang X., Qian S.B. (2011). Chaperone-mediated hierarchical control in targeting misfolded proteins to aggresomes. Mol. Biol. Cell.

[139] Tetzlaff J.E., Putcha P., Outeiro T.F., Ivanov A., Berezovska O., Hyman B.T., McLean P.J. (2008). CHIP targets toxic alpha-synuclein oligomers for degradation. J. Biol. Chem..

[140] Kalia L.V., Kalia S.K., Chau H., Lozano A.M., Hyman B.T., McLean P.J. (2011). Ubiquitinylation of alpha-synuclein by carboxyl terminus Hsp70-interacting protein (CHIP) is regulated by Bcl-2-associated athanogene 5 (BAG5). PLOS ONE.

[141] Su M., Shi J.J., Yang Y.P., Li J., Zhang Y.L., Chen J., Hu L.F., Liu C.F. (2011). HDAC6 regulates aggresome-autophagy degradation pathway of alpha-synuclein in response to MPP^+^-induced stress. J. Neurochem..

[142] Spencer B., Potkar R., Trejo M., Rockenstein E., Patrick C., Gindi R., Adame A., Wyss-Coray T., Masliah E. (2009). Beclin 1 gene transfer activates autophagy and ameliorates the neurodegenerative pathology in alpha-synuclein models of Parkinson’s and Lewy body diseases. J. Neurosci..

[143] Tofaris G.K., Kim H.T., Hourez R., Jung J.W., Kim K.P., Goldberg A.L. (2011). Ubiquitin ligase Nedd4 promotes alpha-synuclein degradation by the endosomal-lysosomal pathway. Proc. Natl. Acad. Sci. USA.

[144] Paxinou E., Chen Q., Weisse M., Giasson B.I., Norris E.H., Rueter S.M., Trojanowski J.Q., Lee V.M., Ischiropoulos H. (2001). Induction of alpha-synuclein aggregation by intracellular nitrative insult. J. Neurosci..

[145] Webb J.L., Ravikumar B., Atkins J., Skepper J.N., Rubinsztein D.C. (2003). Alpha-synuclein is degraded by both autophagy and the proteasome. J. Biol. Chem..

[146] Salvador N., Aguado C., Horst M., Knecht E. (2000). Import of a cytosolic protein into lysosomes by chaperone-mediated autophagy depends on its folding state. J. Biol. Chem..

[147] Sevlever D., Jiang P., Yen S.H. (2008). Cathepsin D is the main lysosomal enzyme involved in the degradation of alpha-synuclein and generation of its carboxy-terminally truncated species. Biochemistry.

[148] Crabtree D., Dodson M., Ouyang X., Boyer-Guittaut M., Liang Q., Ballestas M.E., Fineberg N., Zhang J. (2014). Over-expression of an inactive mutant cathepsin D increases endogenous alpha-synuclein and cathepsin B activity in SH-SY5Y cells. J. Neurochem..

[149] Cullen V., Lindfors M., Ng J., Paetau A., Swinton E., Kolodziej P., Boston H., Saftig P., Woulfe J., Feany M.B. (2009). Cathepsin D expression level affects alpha-synuclein processing, aggregation, and toxicity *in vivo*. Mol. Brain.

[150] Winslow A.R., Chen C.W., Corrochano S., Acevedo-Arozena A., Gordon D.E., Peden A.A., Lichtenberg M., Menzies F.M., Ravikumar B., Imarisio S. (2010). Alpha-synuclein impairs macroautophagy: Implications for Parkinson’s disease. J. Cell Biol..

[151] Crews L., Spencer B., Desplats P., Patrick C., Paulino A., Rockenstein E., Hansen L., Adame A., Galasko D., Masliah E. (2010). Selective molecular alterations in the autophagy pathway in patients with Lewy body disease and in models of alpha-synucleinopathy. PLOS ONE.

[152] Yan J.Q., Yuan Y.H., Gao Y.N., Huang J.Y., Ma K.L., Gao Y., Zhang W.Q., Guo X.F., Chen N.H. (2014). Overexpression of human E46K mutant alpha-synuclein impairs macroautophagy via inactivation of JNK1-Bcl-2 pathway. Mol. Neurobiol..

[153] Choubey V., Safiulina D., Vaarmann A., Cagalinec M., Wareski P., Kuum M., Zharkovsky A., Kaasik A. (2011). Mutant A53T alpha-synuclein induces neuronal death by increasing mitochondrial autophagy. J. Biol. Chem..

[154] Stefanis L., Larsen K., Rideout H. (2001). Expression of A53T mutant but not wild-type α-synuclein in PC12 cells induces alterations of the ubiquitin-dependent degradation system, loss of dopamine release, and autophagic cell death. J. Neurosci..

[155] Braak H., Rüb U., del Tredici K. (2006). Cognitive decline correlates with neuropathological stage in Parkinson’s disease. J. Neurol. Sci..

[156] Braak H., Rüb U., Gai W.P., del Tredici K. (2003). Idiopathic Parkinson’s disease: Possible routes by which vulnerable neuronal types may be subject to neuroinvasion by an unknown pathogen. J. Neural Transm..

[157] Hawkes C.H., Del Tredici K., Braak H. (2009). Parkinson’s disease: The dual hit theory revisited. Ann. NY Acad. Sci..

[158] Kordower J.H., Chu Y., Hauser R.A., Olanow C.W., Freeman T.B. (2008). Transplanted dopaminergic neurons develop PD pathologic changes: A second case report. Mov. Disord..

[159] Brundin P., Li J.-Y., Holton J.L., Lindvall O., Revesz T. (2008). Research in motion: The enigma of Parkinson’s disease pathology spread. Nat. Rev. Neurosci..

[160] Li J.Y., Englund E., Widner H., Rehncrona S., Björklund A., Lindvall O., Brundin P. (2010). Characterization of Lewy body pathology in 12- and 16-year-old intrastriatal mesencephalic grafts surviving in a patient with Parkinson’s disease. Mov. Disord..

[161] Chu Y., Kordower J.H. (2010). Lewy body pathology in fetal grafts. Ann. NY Acad. Sci..

[162] Kurowska Z., Englund E., Widner H., Lindvall O., Li J.Y., Brundin P. (2011). Signs of degeneration in 12–22-year old grafts of mesencephalic dopamine neurons in patients with Parkinson’s disease. J. Parkinsons Dis..

[163] Hansen C., Angot E., Bergström A. (2011). Α-synuclein propagates from mouse brain to grafted dopaminergic neurons and seeds aggregation in cultured human cells. J. Clin. Invest..

[164] Angot E., Steiner J.A., Tomé C.M., Ekström P., Mattsson B., Björklund A., Brundin P. (2012). Alpha-synuclein cell-to-cell transfer and seeding in grafted dopaminergic neurons *in vivo*. PLOS ONE.

[165] Kordower J.H., Dodiya H.B., Kordower A.M., Terpstra B., Paumier K., Madhavan L., Sortwell C., Steece-Collier K., Collier T.J. (2011). Transfer of host-derived alpha synuclein to grafted dopaminergic neurons in rat. Neurobiol. Dis..

[166] Borghi R., Marchese R., Negro A., Marinelli L., Forloni G., Zaccheo D., Abbruzzese G., Tabaton M. (2000). Full length alpha-synuclein is present in cerebrospinal fluid from Parkinson’s disease and normal subjects. Neurosci. Lett..

[167] El-Agnaf O.M.A., Salem S.A., Paleologou K.E., Cooper L.J., Fullwood N.J., Gibson M.J., Curran M.D., Court J.A., Mann D.M.A., Ikeda S. (2003). Alpha-synuclein implicated in Parkinson’s disease is present in extracellular biological fluids, including human plasma. FASEB J..

[168] Paleologou K.E., Kragh C.L., Mann D.M.A., Salem S.A., Al-Shami R., Allsop D., Hassan A.H., Jensen P.H., El-Agnaf O.M.A. (2009). Detection of elevated levels of soluble alpha-synuclein oligomers in post-mortem brain extracts from patients with dementia with lewy bodies. Brain.

[169] Parnetti L., Chiasserini D., Persichetti E., Eusebi P., Varghese S., Qureshi M.M., Dardis A., Deganuto M., de Carlo C., Castrioto A. (2014). Cerebrospinal fluid lysosomal enzymes and alpha-synuclein in Parkinson’s disease. Mov. Disord..

[170] Luk K.C., Kehm V.M., Zhang B., O’Brien P., Trojanowski J.Q., Lee V.M.Y. (2012). Intracerebral inoculation of pathological-synuclein initiates a rapidly progressive neurodegenerative alpha-synucleinopathy in mice. J. Exp. Med..

[171] Mougenot A.L., Nicot S., Bencsik A., Morignat E., Verchère J., Lakhdar L., Legastelois S., Baron T. (2012). Prion-like acceleration of a synucleinopathy in a transgenic mouse model. Neurobiol. Aging.

[172] Luk K.C., Kehm V., Carroll J., Zhang B., O’Brien P., Trojanowski J.Q., Lee V.M.-Y. (2012). Pathological α-synuclein transmission initiates parkinson-like neurodegeneration in nontransgenic mice. Science.

[173] Masuda-Suzukake M., Nonaka T., Hosokawa M., Oikawa T., Arai T., Akiyama H., Mann D.M.A., Hasegawa M. (2013). Prion-like spreading of pathological α-synuclein in brain. Brain.

[174] Recasens A., Dehay B., Bové J., Carballo-Carbajal I., Dovero S., Pérez-Villalba A., Fernagut P.O., Blesa J., Parent A., Perier C. (2014). Lewy body extracts from Parkinson disease brains trigger α-synuclein pathology and neurodegeneration in mice and monkeys. Ann. Neurol..

[175] Guo J.L., Covell D.J., Daniels J.P., Iba M., Stieber A., Zhang B., Riddle D.M., Kwong L.K., Xu Y., Trojanowski J.Q. (2013). Distinct α-synuclein strains differentially promote tau inclusions in neurons. Cell.

[176] Sacino A.N., Brooks M., Thomas M.A., McKinney A.B., McGarvey N.H., Rutherford N.J., Ceballos-Diaz C., Robertson J., Golde T.E., Giasson B.I. (2014). Amyloidogenic α-synuclein seeds do not invariably induce rapid, widespread pathology in mice. Acta Neuropathol..

[177] Recasens A., Dehay B. (2014). Alpha-synuclein spreading in Parkinson disease. Front. Neuroanat..

[178] Costanzo M., Zurzolo C. (2013). The cell biology of prion-like spread of protein aggregates: Mechanisms and implication in neurodegeneration. Biochem. J..

[179] Zetterberg H., Petzold M., Magdalinou N. (2014). Cerebrospinal fluid α-synuclein levels in Parkinson’s disease—Changed or unchanged?. Eur. J. Neurol..

[180] Lee H.-J., Patel S., Lee S.-J. (2005). Intravesicular localization and exocytosis of alpha-synuclein and its aggregates. J. Neurosci..

[181] Danzer K.M., Kranich L.R., Ruf W.P., Cagsal-Getkin O., Winslow A.R., Zhu L., Vanderburg C.R., McLean P.J. (2012). Exosomal cell-to-cell transmission of alpha synuclein oligomers. Mol. Neurodegener..

[182] Emmanouilidou E., Melachroinou K., Roumeliotis T., Garbis S.D., Ntzouni M., Margaritis L.H., Stefanis L., Vekrellis K. (2010). Cell-produced alpha-synuclein is secreted in a calcium-dependent manner by exosomes and impacts neuronal survival. J. Neurosci..

[183] Schneider A., Simons M. (2013). Exosomes: Vesicular carriers for intercellular communication in neurodegenerative disorders. Cell Tissue Res..

[184] Shi M., Liu C., Cook T.J., Bullock K.M., Zhao Y., Ginghina C., Li Y., Aro P., Dator R., He C. (2014). Plasma exosomal α-synuclein is likely CNS-derived and increased in Parkinson’s disease. Acta neuropathol..

[185] Freundt E.C., Maynard N., Clancy E.K., Roy S., Bousset L., Sourigues Y., Covert M., Melki R., Kirkegaard K., Brahic M. (2012). Neuron-to-neuron transmission of a-synuclein fibrils through axonal transport. Ann. Neurol..

[186] Masuda-Suzukake M., Nonaka T., Hosokawa M., Kubo M., Shimozawa A., Akiyama H., Hasegawa M. (2014). Pathological alpha-synuclein propagates through neural networks. Acta Neuropathol. Commun..

[187] Jang A., Lee H.J., Suk J.E., Jung J.W., Kim K.P., Lee S.J. (2010). Non-classical exocytosis of alpha-synuclein is sensitive to folding states and promoted under stress conditions. J. Neurochem..

[188] Lee H.-J., Baek S.M., Ho D.-H., Suk J.-E., Cho E.-D., Lee S.-J. (2011). Dopamine promotes formation and secretion of non-fibrillar alpha-synuclein oligomers. Exp. Mol. Med..

[189] Kondo K., Obitsu S., Teshima R. (2011). Α-synuclein aggregation and transmission are enhanced by leucine-rich repeat kinase 2 in human neuroblastoma SH-SY5Y cells. Biol. Pharm. Bull..

[190] Fares M.B., Ait-Bouziad N., Dikiy I., Mbefo M.K., Jovičić A., Kiely A., Holton J.L., Lee S.J., Gitler A.D., Eliezer D. (2014). The novel Parkinson’s disease linked mutation G51D attenuates *in vitro* aggregation and membrane binding of α-synuclein, and enhances its secretion and nuclear localization in cells. Hum. Mol. Genet..

[191] Khalaf O., Fauvet B., Oueslati A., Dikiy I., Mahul-Mellier A.L., Ruggeri F.S., Mbefo M.K., Vercruysse F., Dietler G., Lee S.J. (2014). The H50Q mutation enhances alpha-synuclein aggregation, secretion, and toxicity. J. Biol. Chem..

[192] Collinge J., Clarke A.R. (2007). A general model of prion strains and their pathogenicity. Science.

[193] Ghosh D., Mondal M., Mohite G.M., Singh P.K., Ranjan P., Anoop A., Ghosh S., Jha N.N., Kumar A., Maji S.K. (2013). The parkinson's disease-associated H50Q mutation accelerates α-synuclein aggregation in vitro. Biochemistry.

[194] Heise H., Celej M.S., Becker S., Riedel D., Pelah A., Kumar A., Jovin T.M., Baldus M. (2008). Solid-state nmr reveals structural differences between fibrils of wild-type and disease-related A53T mutant alpha-synuclein. J. Mol. Biol..

[195] Ono K., Ikeda T., Takasaki J., Yamada M. (2011). Familial Parkinson disease mutations influence alpha-synuclein assembly. Neurobiol. Dis..

[196] Bharathi, Indi S.S., Rao K.S.J. (2007). Copper- and iron-induced differential fibril formation in alpha-synuclein: Tem study. Neurosci. Lett..

[197] Binolfi A., Rasia R.M., Bertoncini C.W., Ceolin M., Zweckstetter M., Griesinger C., Jovin T.M., Fernández C.O. (2006). Interaction of α-synuclein with divalent metal ions reveals key differences: A link between structure, binding specificity and fibrillation enhancement. J. Am. Chem. Soc..

[198] Comellas G., Lemkau L.R., Zhou D.H., George J.M., Rienstra C.M. (2012). Structural intermediates during α-synuclein fibrillogenesis on phospholipid vesicles. J. Am. Chem. Soc..

[199] Hellstrand E., Nowacka A., Topgaard D., Linse S., Sparr E. (2013). Membrane lipid co-aggregation with α-synuclein fibrils. PLOS ONE.

[200] Hoyer W., Antony T., Cherny D., Heim G., Jovin T.M., Subramaniam V. (2002). Dependence of alpha-synuclein aggregate morphology on solution conditions. J. Mol. Biol..

[201] Danzer K.M., Haasen D., Karow A.R., Moussaud S., Habeck M., Giese A., Kretzschmar H., Hengerer B., Kostka M. (2007). Different species of alpha-synuclein oligomers induce calcium influx and seeding. J. Neurosci..

[202] Danzer K.M., Krebs S.K., Wolff M., Birk G., Hengerer B. (2009). Seeding induced by alpha-synuclein oligomers provides evidence for spreading of alpha-synuclein pathology. J. Neurochem..

[203] Bousset L., Pieri L., Ruiz-Arlandis G., Gath J., Jensen P.H., Habenstein B., Madiona K., Olieric V., Böckmann A., Meier B.H. (2013). Structural and functional characterization of two alpha-synuclein strains. Nat. Commun..

[204] Cuanalo-Contreras K., Mukherjee A., Soto C. (2013). Role of protein misfolding and proteostasis deficiency in protein misfolding diseases and aging. Int. J. Cell Biol..

[205] Lee H.J., Cho E.D., Lee K.W., Kim J.H., Cho S.G., Lee S.J. (2013). Autophagic failure promotes the exocytosis and intercellular transfer of alpha-synuclein. Exp. Mol. Med..

[206] Piper R.C., Katzmann D.J. (2007). Biogenesis and function of multivesicular bodies. Annu. Rev. Cell Dev. Biol..

[207] Kong S.M., Chan B.K., Park J.S., Hill K.J., Aitken J.B., Cottle L., Farghaian H., Cole A.R., Lay P.A., Sue C.M. (2014). Parkinson’s disease-linked human PARK9/ATP13A2 maintains zinc homeostasis and promotes alpha-synuclein externalization via exosomes. Hum. Mol. Genet..

[208] Tsunemi T., Hamada K., Krainc D. (2014). ATP13A2/PARK9 regulates secretion of exosomes and alpha-synuclein. J. Neurosci..

[209] Tsunemi T., Krainc D. (2014). Zn^2+^ dyshomeostasis caused by loss of ATP13A2/PARK9 leads to lysosomal dysfunction and alpha-synuclein accumulation. Hum. Mol. Genet..

[210] Leyk J., Goldbaum O., Noack M., Richter-Landsberg C. (2015). Inhibition of HDAC6 modifies Tau inclusion body formation and impairs autophagic clearance. J. Mol. Neurosci. MN.

[211] Fader C.M., Sanchez D., Furlan M., Colombo M.I. (2008). Induction of autophagy promotes fusion of multivesicular bodies with autophagic vacuoles in K562 cells. Traffic.

[212] Poehler A.M., Xiang W., Spitzer P., May V., Meixner H., Rockenstein E., Chutna O., Outeiro T., Winkler J., Masliah E. (2014). Autophagy modulates SNCA/α-synuclein release, thereby generating a hostile microenvironment. Autophagy.

[213] Hruska K.S., LaMarca M.E., Scott C.R., Sidransky E. (2008). Gaucher disease: Mutation and polymorphism spectrum in the glucocerebrosidase gene (GBA). Hum. Mutat..

[214] Ginns E.I., Mak S.K., Ko N., Karlgren J., Akbarian S., Chou V.P., Guo Y., Lim A., Samuelsson S., LaMarca M.L. (2014). Neuroinflammation and alpha-synuclein accumulation in response to glucocerebrosidase deficiency are accompanied by synaptic dysfunction. Mol. Genet. Metab..

[215] Bae E.J., Yang N.Y., Song M., Lee C.S., Lee J.S., Jung B.C., Lee H.J., Kim S., Masliah E., Sardi S.P. (2014). Glucocerebrosidase depletion enhances cell-to-cell transmission of alpha-synuclein. Nat. Commun..

[216] Mazzulli J.R., Xu Y.H., Sun Y., Knight A.L., McLean P.J., Caldwell G.A., Sidransky E., Grabowski G.A., Krainc D. (2011). Gaucher disease glucocerebrosidase and alpha-synuclein form a bidirectional pathogenic loop in synucleinopathies. Cell.

[217] Mantle D., Falkous G., Ishiura S., Perry R.H., Perry E.K. (1995). Comparison of cathepsin protease activities in brain tissue from normal cases and cases with Alzheimer’s disease, Lewy body dementia, Parkinson’s disease and Huntington’s disease. J. Neurol. Sci..

[218] Van Dijk K.D., Persichetti E., Chiasserini D., Eusebi P., Beccari T., Calabresi P., Berendse H.W., Parnetti L., van de Berg W.D. (2013). Changes in endolysosomal enzyme activities in cerebrospinal fluid of patients with Parkinson’s disease. Mov. Disord..

[219] Chu Y., Dodiya H., Aebischer P., Olanow C.W., Kordower J.H. (2009). Alterations in lysosomal and proteasomal markers in Parkinson’s disease: Relationship to alpha-synuclein inclusions. Neurobiol. Dis..

[220] Qiao L., Hamamichi S., Caldwell K.A., Caldwell G.A., Yacoubian T.A., Wilson S., Xie Z.L., Speake L.D., Parks R., Crabtree D. (2008). Lysosomal enzyme cathepsin d protects against alpha-synuclein aggregation and toxicity. Mol. Brain.

[221] Tsujimura A., Taguchi K., Watanabe Y., Tatebe H., Tokuda T., Mizuno T., Tanaka M. (2014). Lysosomal enzyme cathepsin B enhances the aggregate forming activity of exogenous α-synuclein fibrils. Neurobiol. Dis..

[222] Freeman D., Cedillos R., Choyke S., Lukic Z., McGuire K., Marvin S., Burrage A.M., Sudholt S., Rana A., O’Connor C. (2013). Alpha-synuclein induces lysosomal rupture and cathepsin dependent reactive oxygen species following endocytosis. PLOS ONE.

[223] Pratt M.R., Sekedat M.D., Chiang K.P., Muir T.W. (2009). Direct measurement of cathepsin B activity in the cytosol of apoptotic cells by an activity-based probe. Chem. Biol..

[224] Dufty B.M., Warner L.R., Hou S.T., Jiang S.X., Gomez-Isla T., Leenhouts K.M., Oxford J.T., Feany M.B., Masliah E., Rohn T.T. (2007). Calpain-cleavage of alpha-synuclein: Connecting proteolytic processing to disease-linked aggregation. Am. J. Pathol..

[225] Diepenbroek M., Casadei N., Esmer H., Saido T.C., Takano J., Kahle P.J., Nixon R.A., Rao M.V., Melki R., Pieri L. (2014). Overexpression of the calpain-specific inhibitor calpastatin reduces human alpha-synuclein processing, aggregation and synaptic impairment in [A30P]αSyn transgenic mice. Hum. Mol. Genet..

[226] Crocker S.J., Smith P.D., Jackson-Lewis V., Lamba W.R., Hayley S.P., Grimm E., Callaghan S.M., Slack R.S., Melloni E., Przedborski S. (2003). Inhibition of calpains prevents neuronal and behavioral deficits in an mptp mouse model of Parkinson’s disease. J. Neurosci..

[227] Games D., Valera E., Spencer B., Rockenstein E., Mante M., Adame A., Patrick C., Ubhi K., Nuber S., Sacayon P. (2014). Reducing C-terminal-truncated alpha-synuclein by immunotherapy attenuates neurodegeneration and propagation in Parkinson’s disease-like models. J. Neurosci..

[228] Levesque S., Wilson B., Gregoria V., Thorpe L.B., Dallas S., Polikov V.S., Hong J.S., Block M.L. (2010). Reactive microgliosis: Extracellular micro-calpain and microglia-mediated dopaminergic neurotoxicity. Brain.

[229] Rcom-H’cheo-Gauthier A., Goodwin J., Pountney D.L. (2014). Interactions between calcium and α-synuclein in neurodegeneration. Biomolecules.

[230] Reznichenko L., Cheng Q., Nizar K., Gratiy S.L., Saisan P.A., Rockenstein E.M., Gonzalez T., Patrick C., Spencer B., Desplats P. (2012). *In vivo* alterations in calcium buffering capacity in transgenic mouse model of synucleinopathy. J. Neurosci..

[231] Melachroinou K., Xilouri M., Emmanouilidou E., Masgrau R., Papazafiri P., Stefanis L., Vekrellis K. (2013). Deregulation of calcium homeostasis mediates secreted alpha-synuclein-induced neurotoxicity. Neurobiol. Aging.

[232] Yamashima T. (2013). Reconsider Alzheimer’s disease by the “calpain-cathepsin hypothesis”—A perspective review. Prog. Neurobiol..

[233] Syntichaki P., Tavernarakis N. (2003). The biochemistry of neuronal necrosis: Rogue biology?. Nat. Rev. Neurosci..

[234] Hayashi-Nishino M., Fujita N., Noda T., Yamaguchi A., Yoshimori T., Yamamoto A. (2009). A subdomain of the endoplasmic reticulum forms a cradle for autophagosome formation. Nat. Cell Biol..

[235] Bruns C., McCaffery J.M., Curwin A.J., Duran J.M., Malhotra V. (2011). Biogenesis of a novel compartment for autophagosome-mediated unconventional protein secretion. J. Cell Biol..

[236] Dupont N., Jiang S., Pilli M., Ornatowski W., Bhattacharya D., Deretic V. (2011). Autophagy-based unconventional secretory pathway for extracellular delivery of IL-1β. EMBO J..

[237] Koprich J.B., Reske-Nielsen C., Mithal P., Isacson O. (2008). Neuroinflammation mediated by IL-1β increases susceptibility of dopamine neurons to degeneration in an animal model of Parkinson’s disease. J. Neuroinflammation.

[238] Nilsson P., Loganathan K., Sekiguchi M., Matsuba Y., Hui K., Tsubuki S., Tanaka M., Iwata N., Saito T., Saido T.C. (2013). Aβ secretion and plaque formation depend on autophagy. Cell Rep..

[239] Medina D.L., Fraldi A., Bouche V., Annunziata F., Mansueto G., Spampanato C., Puri C., Pignata A., Martina J.A., Sardiello M. (2011). Transcriptional activation of lysosomal exocytosis promotes cellular clearance. Dev. Cell.

[240] Andrews N.W. (2000). Regulated secretion of conventional lysosomes. Trends Cell Biol..

[241] Reddy A., Caler E.V., Andrews N.W. (2001). Plasma membrane repair is mediated by Ca^2+^-regulated exocytosis of lysosomes. Cell.

[242] Andrews N.W. (2005). Membrane repair and immunological danger. EMBO Rep..

[243] Decressac M., Mattsson B., Weikop P., Lundblad M., Jakobsson J., Bjorklund A. (2013). TFEB-mediated autophagy rescues midbrain dopamine neurons from alpha-synuclein toxicity. Proc. Natl. Acad. Sci. USA.

[244] Nemani V.M., Lu W., Berge V., Nakamura K., Onoa B., Lee M.K., Chaudhry F.A., Nicoll R.A., Edwards R.H. (2010). Increased expression of alpha-synuclein reduces neurotransmitter release by inhibiting synaptic vesicle reclustering after endocytosis. Neuron.

[245] Liu S., Ninan I., Antonova I., Battaglia F., Trinchese F., Narasanna A., Kolodilov N., Dauer W., Hawkins R.D., Arancio O. (2004). Alpha-synuclein produces a long-lasting increase in neurotransmitter release. EMBO J..

[246] Tatebe H., Watanabe Y., Kasai T., Mizuno T., Nakagawa M., Tanaka M., Tokuda T. (2010). Extracellular neurosin degrades alpha-synuclein in cultured cells. Neurosci. Res..

[247] Kim K.S., Choi Y.R., Park J.Y., Lee J.H., Kim D.K., Lee S.J., Paik S.R., Jou I., Park S.M. (2012). Proteolytic cleavage of extracellular alpha-synuclein by plasmin: Implications for Parkinson disease. J. Biol. Chem..

[248] Sung J.Y., Park S.M., Lee C.H., Um J.W., Lee H.J., Kim J., Oh Y.J., Lee S.T., Paik S.R., Chung K.C. (2005). Proteolytic cleavage of extracellular secreted α-synuclein via matrix metalloproteinases. J. Biol. Chem..

[249] Park S.M., Kim K.S. (2013). Proteolytic clearance of extracellular alpha-synuclein as a new therapeutic approach against Parkinson disease. Prion.

[250] Lee H.J., Suk J.E., Bae E.J., Lee S.J. (2008). Clearance and deposition of extracellular alpha-synuclein aggregates in microglia. Biochem. Biophys. Res. Commun..

[251] Lee H.J., Suk J.E., Patrick C., Bae E.J., Cho J.H., Rho S., Hwang D., Masliah E., Lee S.J. (2010). Direct transfer of alpha-synuclein from neuron to astroglia causes inflammatory responses in synucleinopathies. J. Biol. Chem..

[252] Deleidi M., Gasser T. (2013). The role of inflammation in sporadic and familial Parkinson’s disease. Cell. Mol. Life Sci..

[253] Garcia-Esparcia P., Llorens F., Carmona M., Ferrer I. (2014). Complex deregulation and expression of cytokines and mediators of the immune response in Parkinson’s disease brain is region dependent. Brain Pathol..

[254] Damier P., Hirsch E.C., Zhang P., Agid Y., Javoy-Agid F. (1993). Glutathione peroxidase, glial cells and Parkinson’s disease. Neuroscience.

[255] Mythri R.B., Venkateshappa C., Harish G., Mahadevan A., Muthane U.B., Yasha T.C., Srinivas Bharath M.M., Shankar S.K. (2011). Evaluation of markers of oxidative stress, antioxidant function and astrocytic proliferation in the striatum and frontal cortex of Parkinson’s disease brains. Neurochem. Res..

[256] Imamura K., Hishikawa N., Sawada M., Nagatsu T., Yoshida M., Hashizume Y. (2003). Distribution of major histocompatibility complex class ii-positive microglia and cytokine profile of Parkinson’s disease brains. Acta Neuropathol..

[257] Mogi M., Harada M., Kondo T., Riederer P., Inagaki H., Minami M., Nagatsu T. (1994). Interleukin-1 beta, interleukin-6, epidermal growth factor and transforming growth factor-alpha are elevated in the brain from parkinsonian patients. Neurosci. Lett..

[258] Halliday G.M., Stevens C.H. (2011). Glia: Initiators and progressors of pathology in Parkinson’s disease. Mov. Disord..

[259] Fellner L., Stefanova N. (2013). The role of glia in alpha-synucleinopathies. Mol. Neurobiol..

[260] Iwai A., Masliah E., Yoshimoto M., Ge N., Flanagan L., de Silva H.A., Kittel A., Saitoh T. (1995). The precursor protein of non-Aβ component of Alzheimer’s disease amyloid is a presynaptic protein of the central nervous system. Neuron.

[261] Wakabayashi K., Hayashi S., Yoshimoto M., Kudo H., Takahashi H. (2000). NACP/α-synuclein-positive filamentous inclusions in astrocytes and oligodendrocytes of Parkinson’s disease brains. Acta Neuropathol..

[262] Hishikawa N., Hashizume Y., Yoshida M., Sobue G. (2001). Widespread occurrence of argyrophilic glial inclusions in Parkinson’s disease. Neuropathol. Appl. Neurobiol..

[263] Braak H., Sastre M., del Tredici K. (2007). Development of alpha-synuclein immunoreactive astrocytes in the forebrain parallels stages of intraneuronal pathology in sporadic Parkinson’s disease. Acta Neuropathol..

[264] Reyes J.F., Rey N.L., Bousset L., Melki R., Brundin P., Angot E. (2014). Alpha-synuclein transfers from neurons to oligodendrocytes. Glia.

[265] Radford R., Rcom-H’cheo-Gauthier A., Wong M.B., Eaton E.D., Quilty M., Blizzard C., Norazit A., Meedeniya A., Vickers J.C., Gai W.P. (2015). The degree of astrocyte activation in multiple system atrophy is inversely proportional to the distance to alpha-synuclein inclusions. Mol. Cell. Neurosci..

[266] Croisier E., Moran L.B., Dexter D.T., Pearce R.K., Graeber M.B. (2005). Microglial inflammation in the parkinsonian substantia nigra: Relationship to alpha-synuclein deposition. J. Neuroinflammation.

[267] Klegeris A., Pelech S., Giasson B.I., Maguire J., Zhang H., McGeer E.G., McGeer P.L. (2008). Alpha-synuclein activates stress signaling protein kinases in Thp-1 cells and microglia. Neurobiol. Aging.

[268] Bae E.J., Lee H.J., Rockenstein E., Ho D.H., Park E.B., Yang N.Y., Desplats P., Masliah E., Lee S.J. (2012). Antibody-aided clearance of extracellular alpha-synuclein prevents cell-to-cell aggregate transmission. J. Neurosci..

[269] Fellner L., Irschick R., Schanda K., Reindl M., Klimaschewski L., Poewe W., Wenning G.K., Stefanova N. (2013). Toll-like receptor 4 is required for alpha-synuclein dependent activation of microglia and astroglia. Glia.

[270] Stefanova N., Fellner L., Reindl M., Masliah E., Poewe W., Wenning G.K. (2011). Toll-like receptor 4 promotes alpha-synuclein clearance and survival of nigral dopaminergic neurons. Am. J. Pathol..

[271] Kim C., Ho D.H., Suk J.E., You S., Michael S., Kang J., Joong Lee S., Masliah E., Hwang D., Lee H.J. (2013). Neuron-released oligomeric alpha-synuclein is an endogenous agonist of TLR2 for paracrine activation of microglia. Nat. Commun..

[272] Park J.Y., Paik S.R., Jou I., Park S.M. (2008). Microglial phagocytosis is enhanced by monomeric alpha-synuclein, not aggregated alpha-synuclein: Implications for Parkinson’s disease. Glia.

